# Highly Selective Enrichment of Glycopeptides Based on Zwitterionically Functionalized Soluble Nanopolymers

**DOI:** 10.1038/srep29776

**Published:** 2016-07-14

**Authors:** Weiqian Cao, Jiangming Huang, Biyun Jiang, Xing Gao, Pengyuan Yang

**Affiliations:** 1 Department of Chemistry, Fudan University, Shanghai 200433, P. R. China; 2 Institutes of Biomedical Sciences, Fudan University, Shanghai 200032, P. R. China

## Abstract

Efficient glycopeptides enrichment prior to mass spectrometry analysis is essential for glycoproteome study. ZIC-HILIC (zwitterionic hydrophilic interaction liquid chromatography) based glycopeptides enrichment approaches have been attracting more attention for several benefits like easy operating, high enrichment specificity and intact glycopeptide retained. In this study, Poly (amidoamine) dendrimer (PAMAM) was adopted for the synthesis of zwitterionically functionalized (ZICF) materials for glycopeptide enrichment. The multiple branched structure and good solubility of ZICF-PAMAM enables a sufficient interaction with glycopeptides. The ZICF-PAMAM combined with the FASP-mode enrichment strategy exhibits more superior performance compared with the existing methods. It has the minimum detectable concentration of femtomolar level and high recovery rate of over 90.01%, and can efficiently enrich glycopeptides from complex biological samples even for merely 0.1 μL human serum. The remarkable glycopeptides enrichment capacity of ZICF-PAMAM highlights the potential application in in-depth glycoproteome research, which may open up new opportunities for the development of glycoproteomics.

Selective enrichment of glycopeptides/glycoproteins prior to mass spectrometry (MS) analysis is a prerequisite for in-depth glycoproteome research^1^, because of the inherent low stoichiometry and microheterogeneity of glycosylation and serious signal suppression of glycopeptides by abundant non-glycopeptides in MS identification^2^. Many ingenious enrichment strategies have been developed based on various principles including hydrazide chemistry (HC), lectin affinity chromatography and hydrophilic interaction chromatography (HILIC). However, to date all the reported enrichment methods have limitations. For example, approaches based on lectin affinity chromatography and HILIC always suffer from low specificity^3^. The HC based strategy although shows good enrichment specificity, the biggest limitation of it lies in that the intact glycopeptides cannot be obtained after enrichment due to the intrinsic enrichment principle of HC^4^. Thus it is still pressing need to develop a glycopeptide enrichment material with high efficiency, good specificity and intact glycopeptides retained for in-depth glycoproteome research.

Recently, zwitterionic hydrophilic interaction liquid chromatography (ZIC-HILIC) as one of the popular HILIC materials has been widely used in glycopeptide isolation^5^. ZIC-HILIC, which is uniquely characterized by carrying both positive and negative charges on surface, has shown better hydrophilic interaction toward glycopeptides than other hydrophilic materials^6^. It can selectively capture glycopeptides from complex samples through hydrophilic and electrostatic interactions, and reversibly release the purified intact glycopeptides. Due to the analytical benefits such as easy operating, short operation time, and higher enrichment specificity, some ZIC-HILIC based materials^7^ have been developed for glycopeptide enrichment. However, the drawbacks of these ZIC-HILIC matrices like limited functional groups and inadequate interaction with glycopeptides in the heterogenous enrichment system significantly restrain the enrichment efficiency toward glycopeptides.

Poly (amidoamine) dendrimer (PAMAM) as a kind of soluble nanno-polymers have been extensively studied since their synthesis in 1985^8^. The merits of structural control, functionalizability and good biocompatibility of PAMAM have made them viable candidates for application in drug delivery^9^, biochemistry and proteome research^10^,^11^,^12^,^13^. Generation 5.0 (G5) PAMAM possesses several attractive properties. First, G5 PAMAM is highly branched with about 128 primary surface amine groups, which can be easily chemically modified and functionalized^14^. Second, the excellent solubility and highly structural homogeneity of PAMAM makes the interaction between materials and glycopeptides completely, which could greatly increase the enrichment sensitivity. Third, G5 PAMAM has a molecular weight of 28826 Da and a theoretical diameter of 5.4 nm. Its molecular weight and size are precisely controlled. Recently, PAMAM was smartly adopted for the synthesis of boronate avidity^15^ and hydrazide functionalized^16^ materials for glycoprotein enrichment. However, boronic acid affinity materials have a common shortcoming of low specificity, while the hydrazide functionalized PAMAM can not retain intact glycopeptides. Therefore, developing a zwitterionically functionalized PAMAM can fully utilize the advantages of dendrimers as well as benefit the intact glycopeptide analysis.

In this work, we propose a chemical strategy for the zwitterionic functionalization (ZICF) of G5 PAMAM for glycopeptide enrichment. With this method we successfully enriched and identified all the glycopeptides from standard glycoproteins with high selectivity and sensitivity. We further applied this strategy to the investigation of human serum glycoproteins and successfully identified 395 unique glycopeptides with 417 glycosylation sites mapped to 178 glycoproteins in three replicates. What’s more, we also identified 44 unique glycopeptides with 48 glycosylation sites from 28 glycoproteins with only 0.1 μL human serum, demonstrating that this strategy can be a powerful tool in the study of protein glycosylation.

## Results and Discussions

**Figure 1:**
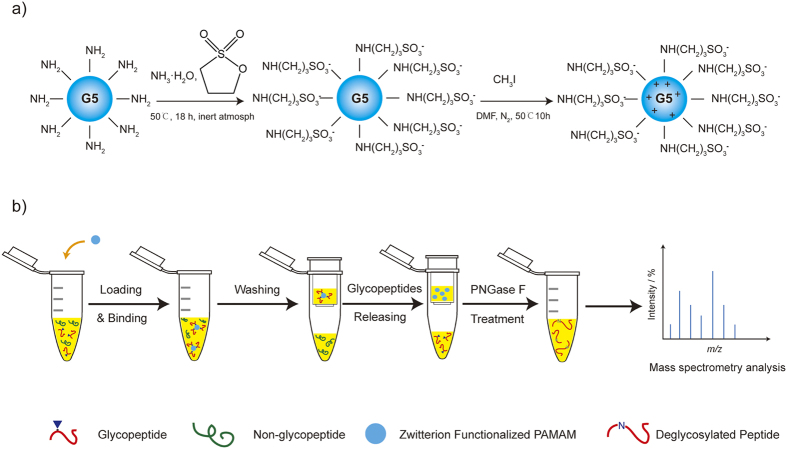
Flowchart of (**a**) Synthesis route for ZICF-PAMAM, and (**b**) ZICF-PAMAM based glycopeptides enrichment with FASP-mode.

The ZICF-PAMAM materials were synthesized based on the commercially available G5 PAMAM via a two-step derivatization procedure according to the previous literatures^17^. The flowchart for the whole synthesis procedure is shown in Fig. 1a. Briefly, G5 PAMAM was sulfonated with 1,3-propanesultone under inert atmosphere. The intermediate then underwent metylation with an excessive amount of iodomethane and puried to finally obtain the ZICF-PAMAM.

**Figure 2:**
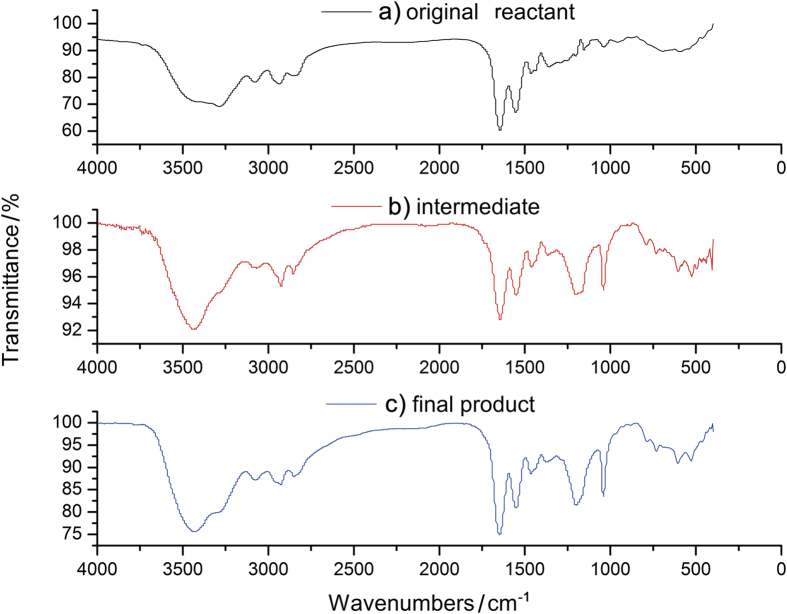
IR spectra of (**a**) the original reactant (G5 PAMAM), (**b**) the intermediate, and (**c**) the final product (ZICF-PAMAM).

**Figure 3:**
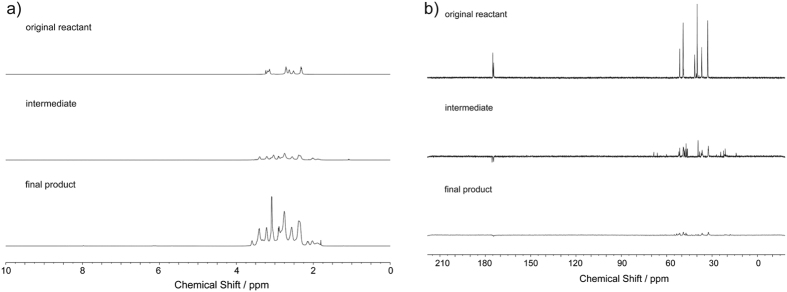
(**a**) ^1^H-NMR (**b**) and ^12^C-NMR spectra of the original reactant (G5 PAMAM, top layer), the intermediate (middle layer), and the final product (ZICF-PAMAM, bottom layer).

To confirm the successful functionalization of the dendrimer, FTIR, H-NMR and C-NMR analysis were employed to analyze the original reactant, intermediate, and final product. The FTIR spectra of three species were shown in Fig. 2. Compared with Fig. 2a (original reactant), there are bands at 1200 and 1040 cm^−1^ belonging to the asymmetric and symmetric SO_3_ H stretching vibration, and the C-S stretch peak at 625 cm^−1^ in Fig. 2b (intermediate) and Fig. 2c (final product), illustrating clearly that the sulfonic acid groups were successfully grafted onto the dendrimer. In Fig. 2c, the enhanced peaks at 1300~1000 cm^−1^ indicates the formation of quaternary ammonium group within the molecular. ^1^H NMR results are shown in Fig. 3a. Signals at δ = 1.8 ppm and δ = 2.55 ppm in Fig. 3a midlle layer (against to Fig. 3a top layer) corresponds to the methylene groups from the reactant 1,3-propanesultone. After methylation, the appearance of the proton signal at δ = 2.2 ppm in Fig. 3a bottom layer indicates the formation of quaternary ammonium groups. The ^13^C-NMR results (Fig. 3b) also confirm the formation of quaternary ammonium groups, for example, the signal at δ = 22 ppm (Fig. 3b midlle layer) is assigned to the methylene groups from the reactant 1,3-propanesultone (contrast to Fig. 3b top layer), and the signals at δ = 53 and 55 ppm appear for the quaternary ammonium groups (Fig. 3b bottom layer). Thus, the above results of structural characterization show fully that the original dendrimers have successfully been zwitterionically functionalized through the two-step derivatization process. In addition, three replicated zeta potential measurements (Table S1 in Supporting Information) demonstrate that the ZICF-PAMAM materials are highly stable in the reaction system, with a surface charge lower than −44.4 mV at a pH 2.0 buffer for glycopeptide enrichment reaction.

**Figure 4:**
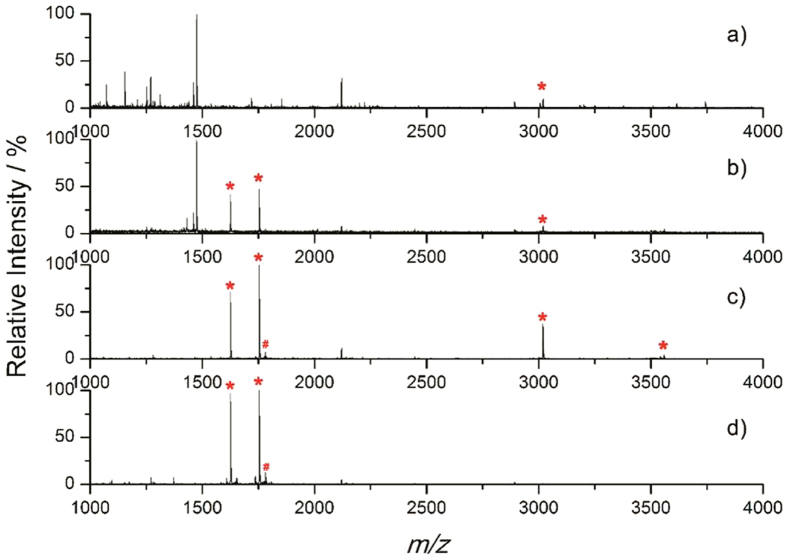
MALDI-TOF spectra of 5 pmol tryptic digests of asialofetuin (**a**) without any pretreatment, (**b**) deglycosylated by PNGase F, (**c**) enriched by ZICF-PAMAM and deglycosylated by PNGase F, (**d**) enriched by commercial Zic-HILIC materials and deglycosylated by PNGase F. (The asterisk denotes the deglycosylated glycopeptides. The pound sign denotes the doubly charged species).

The whole enrichment procedure is illustrated in Fig. 1b. Since the molecular weight and size of PAMAM are precisely controlled. the glycopeptides enriched by the materials can be separated by using a popular sample preparation method in proteome research-filter-aided sample preparation (FASP)^18^. The FASP method can greatly minimize sample loss and eliminate negative influences of interfering substances, thus the enrichment efficiency and specificity could be enhanced. In short, tryptic digested biological samples were incubated with an optimized amount of ZICF-PAMAM. The mixture then underwent ultrafiletration, and the unbound species were descarded. Captured glycopeptides were then released and analyzed with mass spectrometory directly or after deglycosylated with PNGase F. As a proof of concept of this ZICF-PAMAM based strategy, the specificity and enrichment efficiency were firstly investigated by employing asialofetuin tryptic digests as model glycopeptides. As revealed by MALDI-TOF MS spectra analysis (matrix-assisted laser desorption/ionization time-of-flight mass spectrometry), the amount of 5 pmol asialofetuin tryptic digests without any pretreatment are barely detectable among an abundance of non-glycopeptide peaks (Fig. 4a). Although two N-glycosylation sites (Asn156 in LCPDCPLLAPL*N*#DSR or KLCPDCPLLAPL*N*#DSR; Asn176 in VVHAVEVALATFNAES*N*#GSYLQLVEISR) are detected in the spectrum (Fig. 4b) after all linked N-glycans were removed by PNGase F prior to MS analysis, the dominated peaks were still assigned to the non-glycopeptides. However, after enrichment with ZICF-PAMAM, all of the four deglycosylated glycopeptides are observed (Fig. 4c) with high MS intensities and signal-to-noise (S/N) ratios. It is worth mentioning that the intact glycopeptides enriched by ZICF-PAMAM without PNGase F treatment prior to MS analysis can also be evidently detected (Table 1, Fig. S1). Due to the multivalent hydrophilic and electrostatic interactions between the glycans and the positive charged amino and negative charged sulfonic acid groups of ZICF-PAMAM, the glycopeptides can be trapped on the internal and external surface of ZICF-PAMAM. Therefore, the MS intensities and S/N ratios of glycopeptides can be successfully enhanced, indicating the excellent performance of ZICF-PAMAM in selectively enriching glycopeptides.

To further demonstrate such a superior efficiency, two commercial HILIC materials were used for the isolation of glycopeptides as a comparison (Fig. 4d and Fig. S2). Although both intensities and S/N ratios are enhanced compared to Fig. 4b, only two N-glycosylation sites can be confirmed in the spectrum of glycopeptides enriched by commercial amino materials and merely one N-glycosylation sites can be verified in the spectrum of glycopeptides enriched by commercial ZIC-HILIC materials. Moreover, there are still some non-specifically enriched non-glycopeptides in the spectra with relatively high intensity. The incomplete glycopeptide enrichment and the nonspecific binding of commercial materials may be attributed to the following reasons. First, the support matrix of commercial HILIC materials is usually micron-sized silica gel (Fig. S3a) which has high adsorptivity. Thus some non-glycopeptides would be unavoidably absorbed which greatly decrease the enrichment specificity. Second, to enhance the specificity, increasing washing times and bigger washing buffer volume are usually adopted. However, the intensities of enriched glycopeptides would greatly decrease meanwhile. Thus glycopeptides with low-concentration would be lost and some untightly-binding glycopeptides would be randomly washed off during harsh washing procedure, which significantly decrease the enrichment efficiency. Third, the commercial materials have limited functional groups and they present heterogeneous phase in the enrichment system; thus the interaction between material and glycopeptide is inadequate which negatively influence the enrichment efficiency. However, the lost information of glycopeptides and intense nonspecific bindings are both avoided in our enrichment strategy due to the attractive structural characteristics of ZICF-PAMAM: excellent solubility, high structural homogeneity and highly branched with abundant zwitterion functional groups (Fig. S3b). These features enhance the interaction between materials and glycopeptides and make the interaction completely. Moreover, the FASP-mode enrichment procedure greatly minimize the sample loss especially for low abundant glycopeptides, which benefits the enrichment efficiency. The capacity of ZICF-PAMAM for sialylated glycopeptides enrichment was also explored for more general use by using fetuin tryptic digests as an example, which showed that ZICF-PAMAM exihibited more excellent performance in selectively enriching sialyglycopeptides compared with two commercial materials (Fig. S4). Based on the above results and analysis, it is obvious that the ZICF-PAMAM performs superior enrichment efficiency and specificity than the commercial HILIC materials.

**Figure 5:**
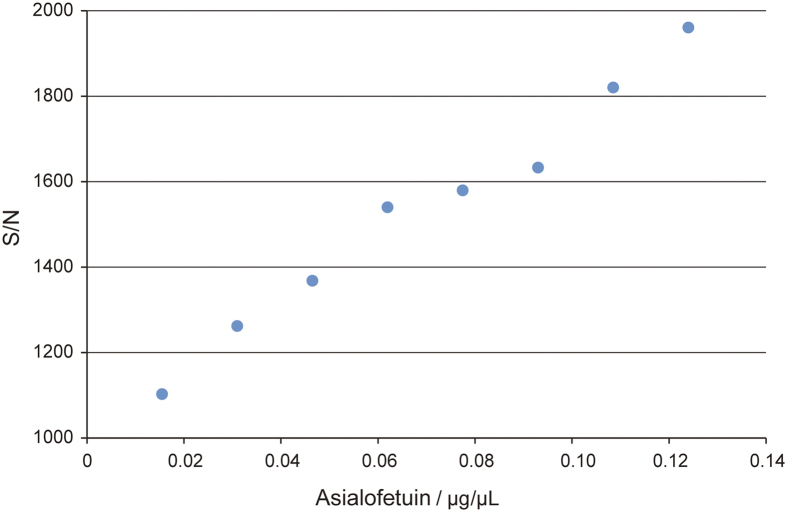
Scatter diagram of the tendency between the S/N value of the N-glycopeptide (KLCPDCPLLAPLN#DSR) and concentration of asialofetuin.

To further evaluate the efficiency of ZICF-PAMAM for selectively enrichment of N-glycopeptides, a series of diluted asialofetuin digests solutions were employed to explore the minimum detectable concentration. This minimum detectable concentration is estimated to be at a level of femtomolar per microliter (Fig. 5 and Fig. S5 in Supporting Information). Then the ZICF-PAMAM based enrichment approach was applied to a much more complex system, a protein mixture of four standard glycoproteins. The results show that all the known N-glycosylation sites with the four glycoproteins have been identified (Table S2 in Supporting Information), which indicate ZICF-PAMAM based strategy is effective and comprehensive for the capture of N-glycopeptides from complex peptide mixtures.

The recovery of ZICF-PAMAM enrichment towards glycopeptides was also evaluated by using asialofetuin tryptic digests and ^18^O labeling technique^19^. The detailed calculation method was shown in S-1 in Supporting Information. The recovery yield of most two abundant glycopeptides from asialofetuin is over 90.01% (Table S3), suggesting that ZICF-PAMAM is an ideal material for the glycopeptides enrichment.

To investigate intensively the feasibility of ZICF-PAMAM for in-depth N-glycosylation analysis, a human serum sample was further employed as a real complex biological one. Highly sensitive and selective enrichment methods for lager-scale glycoproteome analyses of human serum is crucial in diagnostic and therapeutic target discovery. However, the identification and charaterization of human serum glycoproteins remains a challenge for the inherent low concentration of glycoproteins and the dominating high abundant proteins. In this study, a volume of 1 μL human serum was applied to our enrichment strategy. After three replicated analysis with LC-MS/MS, a total of 395 unique glycopeptides with 417 glycosylation sites mapped to 178 glycoproteins have been successfully identified (Table S4 in Supporting Information). The reproducibility of identified N-glycoproteins, glycopeptides and glycosylated sites between three replicates were shown in Fig. S6. It was found that about 82.6% glycoproteins (147 out of 178) were identified at least in two replicates; 82.5% glycopeptides (326 out of 395 ) and 83.0% sites (346 out of 417) were detected at least in two replicates, indicating ZICF-PAMAM exhibiting excellent glycopeptide capture performance for comprehensive glycosylation exploration in complex biological samples. Among the 417 identified N-glycosylated sites, 68 sites were novel sites from this experiment, including 33 sites annotated as“manual assertion according to rules” or “manual assertion inferred from sequence similarity” and 35 sites totally unknown in the latest Uniprot database. Moreover, it is worth mentioning that trace amount of 0.1 μL human serum sample was also applied to our experiment strategy. It should be note that still a total of 44 unique glycopeptides with 48 glycosylation sites mapped to 28 glycoproteins can be identified from such a small volume of human serum within one LC-MS/MS run only (Table S5 in Supporting Information). The mass spectra of two N-glycopeptides identified from 0.1 μL human serum sample were shown in Fig. S7 as examples. The above results indicate that ZICF-PAMAM based enrichment strategy shows extreme efficiency and superior sensitivity.

In summary, a novel ZICF-PAMAM has been successfully designed and synthesized, which bears a number of attractive features: excellent solubility, high structural homogeneity and highly branched with abundant zwitterion functional groups. ZICF-PAMAM combined with the FASP-mode enrichment strategy exhibits remarkable performance for highly selective and sensitive N-glycopeptide enrichment and for comprehensive glycosylation exploration in complex biological samples. Thus the proposed ZICF-PAMAM strategy highlights the potential application in in-depth glycoproteome research, which may open up new opportunities for the development of glycoproteomics.

## Methods

### Chemicals and Reagents

Fetuin and asialofetuin from bovine serum, Ribonuclease B from bovine pancreas, Albumin from chicken egg white, IgG from human serum, PAMAM dendrimer (ethylenediamine core, generation 5.0 solution, provided as 5 wt. % in methanol), 1,3-Propanesultone, dimethylformamide(DMF) were purchased from Sigma (St. Louis, MO, USA). Sequencing grade trypsin was purchased from Promega Corporation (Madison, USA). PNGase F (glycerol free) was purchased from New England Biolabs (Ipswich, MA). H_2_^18^O (97.8%) was purchased from Cambridge Isotope Laboratories (Andover, MA). The 10,000 Da MWCO centrifugal filters were purchased from Millipore (Bedford, MA), and porous graphitized carbon (PGC) columns were purchased from Grace (Columbia, MD). Pure water was prepared with a Milli-Q system (Millipore, Bedford, MA, USA). Iodomethane(CH_3_I), ethanol(CH_3_CH_2_OH), acetone(CH_3_COCH_3_) and all other chemicals and reagents were of analytical grade and obtained from Shanghai Chemical Reagent.

The human serum sample was kindly provide by Fudan University Shanghai Zhongshan hospital. Informed consent was gained from each participant. The research followed the tenets of the Declaration of Helsinki and was approved by the Ethics Committee of the Fudan University Shanghai Zhongshan hospital.

### Synthesis and Characterization of ZICF-PAMAM

G5 PAMAM (2 mL, 5 w.t. % in methanol) was added into a 25 mL round-bottomed flask, rotary evaporated and then redissolved with 2 mL ethanol. The flask was evacuated and back-filled with N_2_. Afterwards, 28% ammonium hydroxide (22 μL) and 1,3-propanesultone (31 μL) in 2 mL ethanol were slowly added, and then stirred at 50 °C for 24 h, which resulted in the formation of intermediate of white precipitates. The white precipitates were washed with ethanol for three times, rotary evaporated and redissolved with 1 mL DMF. Then, anhydrous sodium carbonate (0.17726 g) was added into the flask. The flask was evacuated and back-filled with N_2_. After that, iodomethane (100 μL) in 1 mL DMF was slowly added, and stirred at 50 °C for 12 h. The DMF was removed via lyophilization. A total of 2 mL ethanol/acetone (1:10, v/v) was added to precipitate out a pale-yellow crude product. The crude product was finally purified through dialysis for two days to obtain a colourless, transparent and water-soluble solid, which is the finally product. Fourier-transform infrared (FT-IR) spectra were collected on a Nicolet Fourier spectrophotometer, using KBr pellets. ^1^H NMR and ^13^C-NMR were carried out on a Bruker BioSpin GmbH 500 NMR spectrometer (400 MHz, 298 K) with D_2_O as the solvent. Zeta-potential test was done on a Malvern Zetasizer Nano ZS90.

### Preparation of Protein Digests

Standard glycoprotein (fetuin, asialofetuin, ovalbumin, IgG, ribonuclease B) was dissolved in 50 mM ammonium bicarbonate solution (pH 8.5) at a concentration of 1 μg/μL and heated at 95 °C for 5 min. After cooling to room temperature, the proteins solution was digested with trypsin at37 °C (enzyme to protein mass ratio of 1:50, w/w) for 16 h, and then heated at 95 °C for 5 min to stop the digestion. The tryptic digests were dried through vacuum centrifugation and stored at −20 °C until used. For human serum sample, the serum was diluted in 50 mM ammonium bicarbonate solution (pH 8.5) and denatured by adding 8 mM urea, 10 mM dithiothreitol at 37 °C for 1 h, followed by 20 mM iodoacetamide at room temperature for 30 min in dark. The solution was diluted until the urea concentration reached 1 mM and treated with typsin at 37 °C (enzyme to protein mass ratio of 1:50, w/w) for 16 h. The tryptic digests were desalted using C_18_- SPE, lyophilized through vacuum centrifugation and stored at −20 °C until used.

### Enrichment of N-Glycopeptides with ZICF-PAMAM

Tryptic digests of protein were redissolved in binding buffer (80% ACN-H_2_O, 0.1% TFA) at a concentration of 1 μg/μL. Then the synthesized materials were added (protein/material ratio of 1:40, w/w) and incubated at room temperature for 5 min with continuous shaking. After the binding reaction, the reaction solution was transferred into a 10 kDa MWCO filter and the non-bound peptides were remove into the filtrate collection tube through centrifugation (14000 g) at 18 °C for 8 min. Then the glycopeptides-bound material was washed by washing buffer (80% ACN-H_2_O, 0.5% TFA) through centrifugation (14000 g) at 18 °C for 8 min for three times. Afterwards, the filter was transferred onto a new collection tube and the elution buffer (1% TFA) was added. The glycopeptides were eluted by elution buffer through centrifugation (14000 g) at 18 °C for 8 min for three times. The glycopeptides solution was collected into the new collection tube while the materials remained in the filter.

### Deglycosylation of N-Glycopeptides with PNGase F

The glycopeptides were dissolved in 25 mM ammonium bicarbonate solution (pH 8.5) at a concentration of 0.5–1 μg/μL and treated with PNGase F (500 units per μL) at a concentration of 1 μL PNGase F/1 mg proteins at 37 °C for 16 h. The reaction was stopped by heating to 95 °C for 5 min. Then the glycopeptide solution was lyophilized through vacuum centrifugation and stored at −20 °C until used.

### Mass Spectrometry Analysis

All MALDI-TOF MS experiments were carried out on AB Sciex 5800 MALDI-TOF-TOF mass spectrometry (AB Sciex, CA) in a reflector positive mode with the UV laser at a 400 Hz repetition rate and wavelength of 355 nm. The sample was resuspended with 30% ACN containing 0.1%TFA and spotted onto a MALDI target plate. After air drying, the sample spots were overlaid with 1 μL of CHCA matrix (a-cyano-4-hydroxycinnamic acid, 10 mg/mL in 50% ACN-H_2_O containing 0.1% TFA) for deglycosylated peptides or with 1 μL of DHB matrix (2,5-dihydroxybenzoicacid, 50 mg/mL in 100% ethanol) for glycopeptides, and then analyzed by 5800 MALDI-TOF. Prior to analysis, myoglobin digests were used to calibrate the mass spectrometer with an internal calibration mode.

The nano LC-MS/MS analysis was performed on a Triple TOF 5600 mass spectrometer (AB Sciex, CA) with a nano-HPLC (Eksigent Technologies). The peptides were suspended in 5% (v/v) ACN containing 0.1% (v/v) FA (phase A), separated by a 15-cm reverse phase column with a gradient of 5–45% phase B (95% ACN with 0.1% FA) over 120 min. The peptides were analyzed by MS and data-dependent MS/MS acquisition. MS spectra were acquired across the mass range of 350–1250 m/z in high resolution mode using 250 ms accumulation time per spectrum. Tandem mass spectra scanned from 100–1250 m/z in high sensitivity mode with rolling collision energy. The 20 most intense precursors were selected for fragmentation per cycle with dynamic exclusion time of 9 s.

### Database Searching and Data Analysis

The raw data was initially converted into MGF format with MM File conversion software (Version 3.9). The acquired MS/MS spectra were searched against Swiss-Prot database using MASCOT software (version 2.3). The searching parameters were set as follows: fixed modification of cysteine residues (C, +57 Da), variable modifications of methionine oxidation (M, +16 Da) and deamidation (N, +0.98 Da), two missed tryptic cleavage sites, 20 ppm error tolerance in MS and 0.1 Da error tolerance in MS/MS. The cut-off false discovery rate for all peptide identification was controlled bellow 1%. Only peptides with N–X-S/T(XP)) sequon were considered as N-glycopeptides.

## Additional Information

**How to cite this article**: Cao, W. *et al*. Highly Selective Enrichment of Glycopeptides Based on Zwitterionically Functionalized Soluble Nanopolymers. *Sci. Rep.* **6**, 29776; doi: 10.1038/srep29776 (2016).

## Supplementary Material

Supplementary Information

## Figures and Tables

**Table 1:**
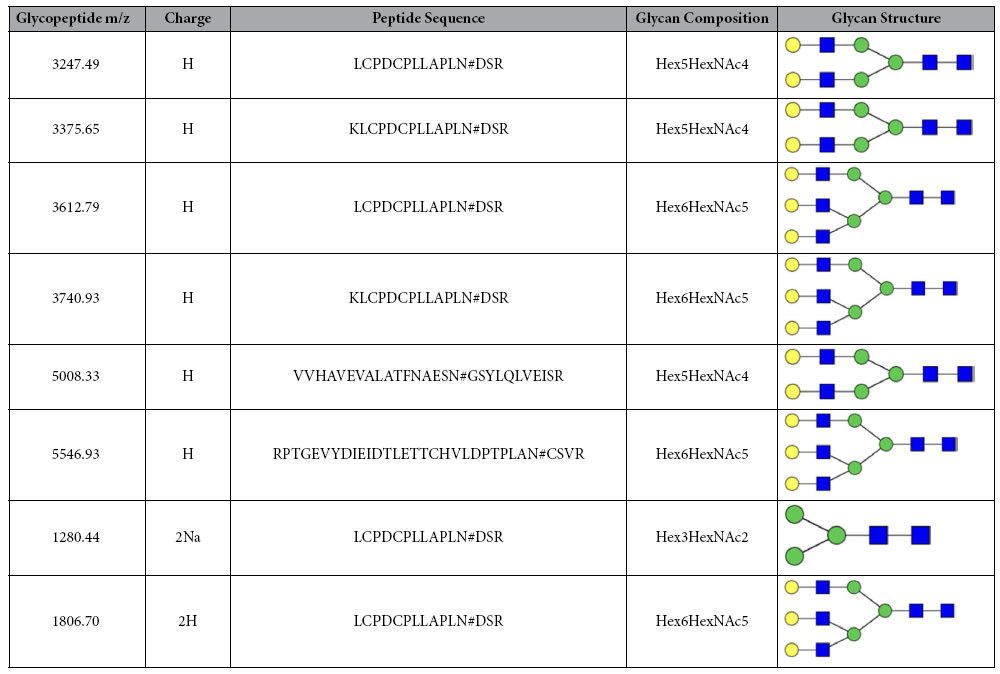
List of identified intact glycopeptides from asialofetuin enriched by ZICF-PAMAM.
