# Supplementary material for: Highly Selective Enrichment of Glycopeptides Based on Zwitterionically Functionalized Soluble Nanopolymers
Source: Sci Rep. 2016 Jul 14;6:29776. doi: 10.1038/srep29776 (PMC4944192; doi:10.1038/srep29776)

Supporting Information

Highly Selective Enrichment of Glycopeptides Based on Zwitterionically Functionalized Soluble Nanopolymers

*Weiqian Cao, ‡ Jiangming Huang, ‡ Biyun Jiang, Xing Gao and Pengyuan Yang**

Table S1. Three replicated zeta potential measurements of the ZICF-PAMAM.

Replicates	*T*/°C	*Zeta potential*/mV
1	24.9	39.2
2	25.0	38.2
3	25.1	44.4

Figure S1. MALDI-TOF spectra of 5 pmol tryptic digests of asialofetuin enriched by ZICF-PAMAM and without PNGase F treatment. (The plus sign denotes glycopeptides.)

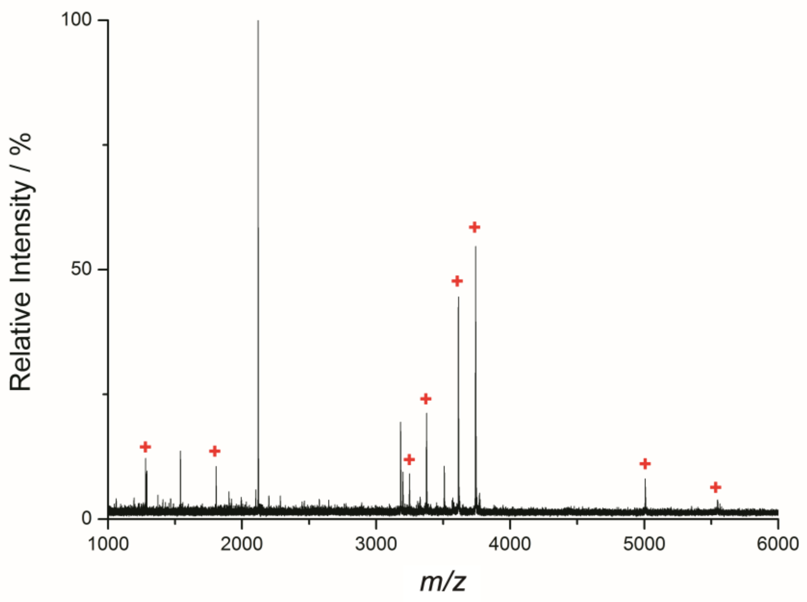

‡: These authors contributed equally.

Figure S2. MALDI-TOF spectra of 5 pmol tryptic digests of asialofetuin enriched by commercial amino materials. (The asterisk denotes the deglycosylated glycopeptide. The pound sign denotes the doubly charged species.)

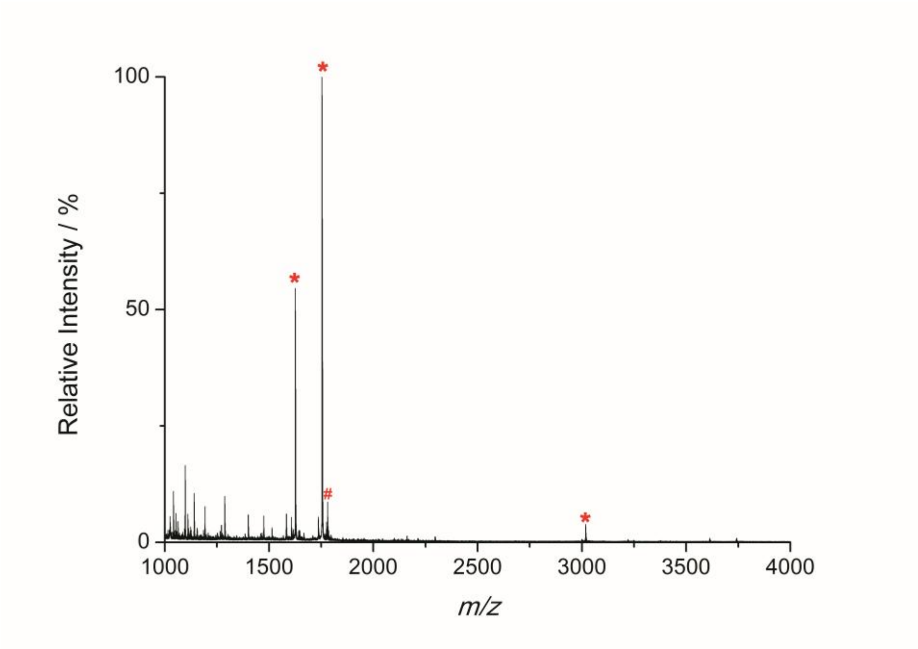

Figure S3. Chemical structures and enrichment principles of commercial materials a) and ZICF-PAMAM b).

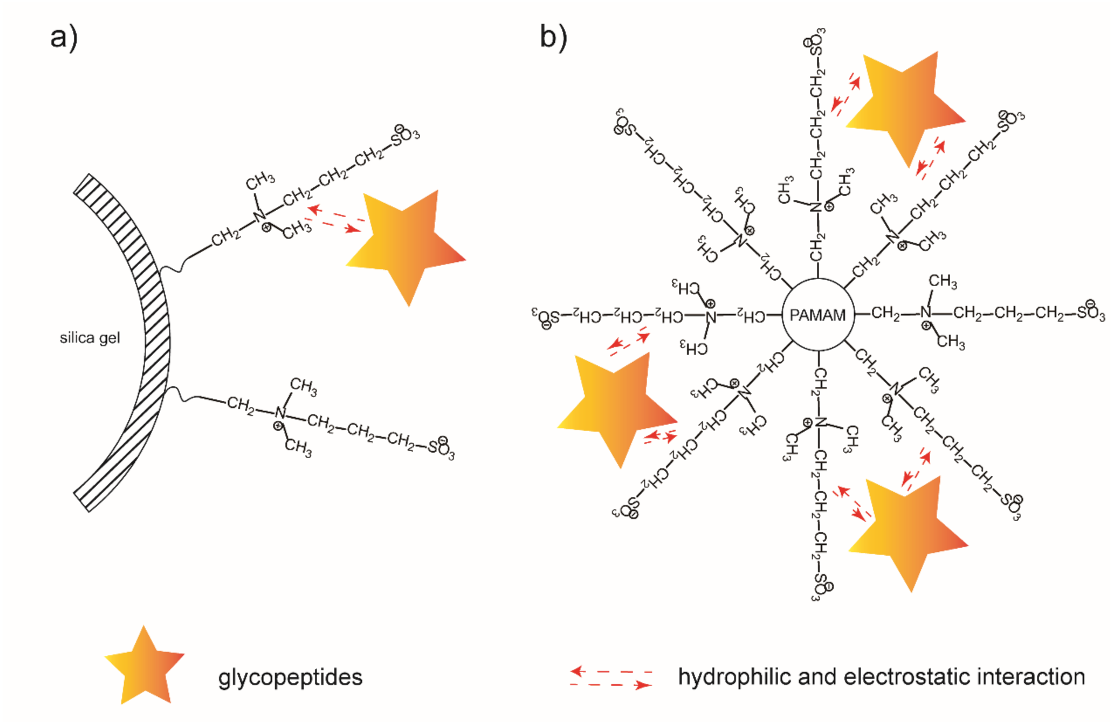

Figure S4. MALDI-TOF spectra of 5 pmol tryptic digests of bovine fetuin a) deglycosylated by PNGase F, b) enriched by ZICF-PAMAM and deglycosylated by PNGase F, c) enriched by commercial ZIC-HILIC materials and deglycosylated by PNGase F. d) enriched by commercial amino materials and deglycosylated by PNGase F. (The asterisk denotes the deglycosylated glycopeptides. The pound sign denotes the doubly charged species.)

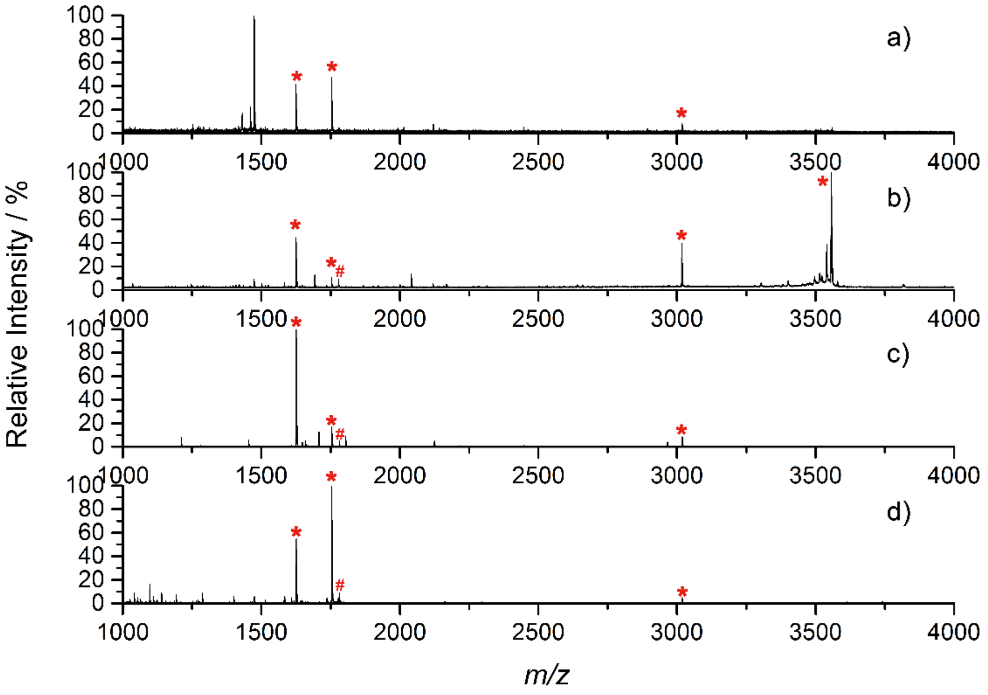

Figure S5. MALDI-TOF MS spectrum of analysis of asialofetuin at a concentration of 0.0155 μg/μL after enrichment with ZICF-PAMAM then deglycosylated by PNGase F. (The asterisk denotes the deglycosylated glycopeptide and the pound sign denotes the doubly charged species).

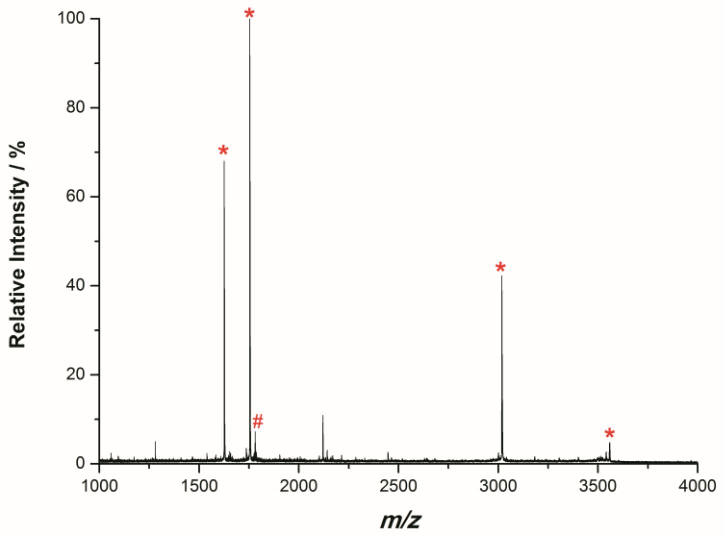

Table S2 Results of LC-MS/MS analysis of N-glycopeptides isolated from the four-glycoproteins mixture.

Standard glycoprotein	Sequences of identified glycoproteins	Theoretical glycosylation sites	Identified glycosylation sites
Fetuin	K.LCPDCPLLAPLN#DSR.V<br>R.KLCPDCPLLAPLN#DSR.V<br>R.VVHAVEVALATFNAESN#GSYLQLVEISR.A<br>R.RPTGEVYDIEIDTLETTCHVLDPTPLAN#CSVR.Q	3	3
Ovalbumin	K.WTNDLGSN#MTIGAVNSR.G	1	1
IgG	R.EEQFN#STFR.V<br>K.TKPREEQYN#STYR.V<br>R.EEQYN#STYR.V	2	2
RNB	K.SRN#LTKDR.C	1	1

N# denotes the N-glycosylation sites.

S-1. Calculation of the enrichment recovery of ZICF-PAMAM towards glycopeptides

The enrichment recovery was evaluated by our previously developed ^{18}O labeling technique (*ACS Appl. Mater. Interfaces* ***5,*** 614-621 (2013)) with a little modification. Briefly tryptic digested peptides of asialofetuin (ASF) were divided into two of equal amount. One of them was enriched by ZICF- PAMAM and released by PNGase F in 50 mM ammonium bicarbonate solution prepared by $H_2{}^{16}O$. The other one was directly treated by PNGase F in 50 mM ammonium bicarbonate solution prepared by $H_2{}^{18}O$. Then the two samples were mixed and analyzed by MALDI-MS.

Taken into account the influence of isotope peaks and labeling efficiency, a self-conducted equation was used to obtain the accurate recovery rate of our material. The calculated equation is as follows:

$$recovery\ rate = \frac{I_{\mathrm{A}} - \mathrm{r}_a I_{\mathrm{B}}}{I_{\mathrm{B}} - \mathrm{r}_b I_{\mathrm{A}}}$$

I_{A}: intensity of monoisotope peak of ASF glycopeptides in the sample enriched by ZICF-PAMAM;

I_{B}: intensity of 2 Da higher peak in the ^{18}O labeled sample corresponding to the same glycopeptide of ASF without enrichment;

r_a: ratio of the labeling efficiency for enzymatic ^{18}O labeling method. The r_a was calculated to be 0.11 by experiment;

r_b: ratio of 2 Da higher peak against monoisotope peak of glycopeptides. The r_b was calculated by a web-based tool called *MS-Isotope (http://prospector.ucsf.edu/prospector/cgi-bin/msform.cgi?form=msisotope)* using each peptide sequence. For peptide LCPDCPLLAPL*N*DSR, r_b equals to 0.5194, and for peptide KLCPDCPLLAPL*N*DSR, r_b equals to 0.5918.

Table S3 Recovery of two of most abundance glycopeptides from asialofetuin digests after enrichment by ZICF-PAMAM.

Glycopeptide sequence	Observed peptide m/z(after enrichment)	Observed peptide m/z(before enrichment)	Peak area after enrichment	Peak area before enrichment	Recovery rate(%)	STD(%)	Averaged recovery rate(%)
LCPDCPLLAPLNDSR	1625.80	1627.80	8869.8	12802.7	91.04		
LCPDCPLLAPLNDSR	1625.85	1627.84	6894.1	10044.7	89.56	0.77	90.42
LCPDCPLLAPLNDSR	1625.86	1627.86	7585.9	10975.3	90.67		
KLCPDCPLLAPLNDSR	1753.91	1755.91	2581.6	3814.5	94.55		
KLCPDCPLLAPLNDSR	1753.96	1955.96	1917.6	2932.2	88.74	4.06	90.01
KLCPDCPLLAPLNDSR	1753.97	1755.97	1907.5	2953.7	86.72		

Table S4 List of identified glycoproteins and glycopeptide sequences from tryptic digests of 1 µL human plasma enriched by ZICF-PAMAM and analyzed by LC-MS/MS with three replicates.

Protein accession number	Protein description	Peptide sequence	Sites annotated in Uniprot
P01009	Alpha-1-antitrypsin OS=Homo sapiens GN=SERPINA1 PE=1 SV=3	QLAHQSN#STNIFFSPVSIATAFAMLSLGTK	known
P01009	Alpha-1-antitrypsin OS=Homo sapiens GN=SERPINA1 PE=1 SV=3	ADTHDEILEGLNFN#LTEIPEAQIHEGFQELLR	known
P01023	Alpha-2-macroglobulin OS=Homo sapiens GN=A2M PE=1 SV=3	SLGNVN#FTVSAEALESQELCGTEVPSVPEHGR	known
P01023	Alpha-2-macroglobulin OS=Homo sapiens GN=A2M PE=1 SV=3	GNEANYYSNATTDEHGLVQFSIN#TTNVMGTSLTVR	known
P01024	Complement C3 OS=Homo sapiens GN=C3 PE=1 SV=2	TVLTPATNHMGN#VTFTIPANR	known

P04114	Apolipoprotein B-100 OS=Homo sapiens GN=APOB PE=1 SV=2	FN#SSYLQGTNQITGR	known
P04114	Apolipoprotein B-100 OS=Homo sapiens GN=APOB PE=1 SV=2	VNQNLVYESGSLN#FSK	known
P04114	Apolipoprotein B-100 OS=Homo sapiens GN=APOB PE=1 SV=2	QVFPGLN#YCTSGAYSN#ASSTDSASYYPLTGDTR	unknown;prediction
P01011	Alpha-1-antichymotrypsin OS=Homo sapiens GN=SERPINA3 PE=1 SV=2	TLN#QSSDELQLSMGNAMFVK	known
P00738	Haptoglobin OS=Homo sapiens GN=HP PE=1 SV=1	NLFLN#HSEN#ATAK	known;known
P00738	Haptoglobin OS=Homo sapiens GN=HP PE=1 SV=1	MVSHHN#LTTGATLINEQWLLTTAK	known
P01871	Ig mu chain C region OS=Homo sapiens GN=IGHM PE=1 SV=3	YKN#NSDISSTR	known
P01871	Ig mu chain C region OS=Homo sapiens GN=IGHM PE=1 SV=3	THTN#ISESHPN#ATFSAVGEASICEDDWNSGER	known;known
P02763	Alpha-1-acid glycoprotein 1 OS=Homo sapiens GN=ORM1 PE=1 SV=1	QDQCIYN#TTYLNVQR	known
P02787	Serotransferrin OS=Homo sapiens GN=TF PE=1 SV=3	CGLVPVLAENYN#K	known
P02787	Serotransferrin OS=Homo sapiens GN=TF PE=1 SV=3	QQQHLFGSN#VTDCSGN#FCLFR	known;unknown
P00450	Ceruloplasmin OS=Homo sapiens GN=CP PE=1 SV=1	ELHHLQEQN#VSNAFLDK	known
P00450	Ceruloplasmin OS=Homo sapiens GN=CP PE=1 SV=1	ELHHLQEQN#VSNAFLDKGEFYIGSK	known
P08603	Complement factor H OS=Homo sapiens GN=CFH PE=1 SV=4	MDGASN#VTCINSR	known
P08603	Complement factor H OS=Homo sapiens GN=CFH PE=1 SV=4	SPYEMFGDEEVMCLNGN#WTEPPQCK	known
P08603	Complement factor H OS=Homo sapiens GN=CFH PE=1 SV=4	WDPEVN#CSMAQIQLCPPPPQIPNSHNMTTTLNYR	known
P08603	Complement factor H OS=Homo sapiens GN=CFH PE=1 SV=4	LN#DTLDYECHDGYESNTGSTTGSIVCGYNGWSDLPICYER	known
P02790	Hemopexin OS=Homo sapiens GN=HPX PE=1 SV=2	N#GTGHGN#STHHGPEYMR	known;known
P19652	Alpha-1-acid glycoprotein 2 OS=Homo sapiens GN=ORM2 PE=1 SV=2	QNQCFYN#SSYLNVQR	known

P02765	Alpha-2-HS-glycoprotein OS=Homo sapiens GN=AHSG PE=1 SV=1	AALAAFNAQNN#GSNFQLEEISR	known
P01042	Kininogen-1 OS=Homo sapiens GN=KNG1 PE=1 SV=2	LNAENN#ATFYFK	known
P01042	Kininogen-1 OS=Homo sapiens GN=KNG1 PE=1 SV=2	HGIQYFNN#NTQHSSLFMLNEVK	known
P05090	Apolipoprotein D OS=Homo sapiens GN=APOD PE=1 SV=1	CIQAN#YSLMENGK	known
P05090	Apolipoprotein D OS=Homo sapiens GN=APOD PE=1 SV=1	ADGTVNQIEGEATPVN#LTEPAK	known
P25311	Zinc-alpha-2-glycoprotein OS=Homo sapiens GN=AZGP1 PE=1 SV=2	FGCEIENN#R	known
P25311	Zinc-alpha-2-glycoprotein OS=Homo sapiens GN=AZGP1 PE=1 SV=2	DIVEYYN#DSN#GSHVLQGR	known;known
P25311	Zinc-alpha-2-glycoprotein OS=Homo sapiens GN=AZGP1 PE=1 SV=2	GDVLHNGN#GTYQSWVVVAVPPQDTAPYSCHVQHSSLAQPLV VPWEAS	known
P04220	Ig mu heavy chain disease protein OS=Homo sapiens PE=1 SV=1	THTN#ISESHPN#ATFSAVGEASICEDDWDSGER	unknown;known
P02749	Beta-2-glycoprotein 1 OS=Homo sapiens GN=APOH PE=1 SV=3	VYKPSAGN#NSLYR	known
P02749	Beta-2-glycoprotein 1 OS=Homo sapiens GN=APOH PE=1 SV=3	DTAVFECLPQHAMFGN#DTITCTTHGN#WTK	known;known
P04004	Vitronectin OS=Homo sapiens GN=VTN PE=1 SV=1	NN#ATVHEQVGGPSLTSDLQAQSK	known
P10909	Clusterin OS=Homo sapiens GN=CLU PE=1 SV=1	MLN#TSSLLEQLNEQFNWVSR	known
O75882	Attractin OS=Homo sapiens GN=ATRN PE=1 SV=2	IDSTGN#VTNELR	known
O75882	Attractin OS=Homo sapiens GN=ATRN PE=1 SV=2	YN#WSFIHCPACQCNGHSK	prediction
O75882	Attractin OS=Homo sapiens GN=ATRN PE=1 SV=2	AATCINPLN#GSVCERPAN#HSAK	prediction;predict ion
P20851	C4b-binding protein beta chain OS=Homo sapiens GN=C4BPB PE=1 SV=1	LGHCPDPVLVNGEFSSSGPVN#VSDK	known
P04003	C4b-binding protein alpha chain OS=Homo sapiens GN=C4BPA PE=1 SV=2	LSVDKDQYVEPEN#VTIQCDSGYGVVGPQSITCSGN#R	known;known
P04196	Histidine-rich glycoprotein OS=Homo sapiens GN=HRG PE=1 SV=1	HSHNN#N#SSDLHPHK	known;unknown

P04196	Histidine-rich glycoprotein OS=Homo sapiens GN=HRG PE=1 SV=1	VIDFN#CTTSSVSSALANTK	known
P06681	Complement C2 OS=Homo sapiens GN=C2 PE=1 SV=2	LTDTICGVGN#MSAN#ASDQER	known;known
P04217	Alpha-1B-glycoprotein OS=Homo sapiens GN=A1BG PE=1 SV=4	FQSPAGTEALFELHN#ISVADSAN#YSCVYVDLKPPFGGSAPSER	known;known
P00734	Prothrombin OS=Homo sapiens GN=F2 PE=1 SV=2	N#FTENDLLVR	known
P00734	Prothrombin OS=Homo sapiens GN=F2 PE=1 SV=2	YPHKPEIN#STTHPGADLQEN#FCR	known;unknown
P05156	Complement factor I OS=Homo sapiens GN=CFI PE=1 SV=2	FLNN#GTCTAEGK	known
P02751	Fibronectin OS=Homo sapiens GN=FN1 PE=1 SV=4	LDAPTNLQFVN#ETDSTVLVR	known
P02751	Fibronectin OS=Homo sapiens GN=FN1 PE=1 SV=4	GGNSNGALCHFPFLYNNHN#YTDCTSEGR	known
P43652	Afamin OS=Homo sapiens GN=AFM PE=1 SV=1	DIENFN#STQK	known
P43652	Afamin OS=Homo sapiens GN=AFM PE=1 SV=1	N#CCNTENPPGCYR	known
P49908	Selenoprotein P OS=Homo sapiens GN=SEPP1 PE=1 SV=3	EGYSN#ISYIVVNHQGISSR	known
P49908	Selenoprotein P OS=Homo sapiens GN=SEPP1 PE=1 SV=3	VSEHIPVYQQEEN#QTDVWTLLN#GSK	known;known
P01833	Polymeric immunoglobulin receptor OS=Homo sapiens GN=PIGR PE=1 SV=4	QIGLYPVLVIDSSGYVNPN#YTGR	known
P01833	Polymeric immunoglobulin receptor OS=Homo sapiens GN=PIGR PE=1 SV=4	ANLTNFPEN#GTFVVNIAQLSQDDSGR	known
P07358	Complement component C8 beta chain OS=Homo sapiens GN=C8B PE=1 SV=3	LLCNGDN#DCGDQSDEANCR	unknown
O75636	Ficolin-3 OS=Homo sapiens GN=FCN3 PE=1 SV=2	VELEDFNGN#R	known
Q03591	Complement factor H-related protein 1 OS=Homo sapiens GN=CFHR1 PE=1 SV=2	LQNNENN#ISCVER	known
Q03591	Complement factor H-related protein 1 OS=Homo sapiens GN=CFHR1 PE=1 SV=2	SPYEMFGDEEVMCLNGN#WTEPPQCK	known
P19827	Inter-alpha-trypsin inhibitor heavy chain H1 OS=Homo sapiens GN=ITIH1 PE=1 SV=3	DKICDLLVANNHFAHFFAPQN#LTNMNK	known

P51884	Lumican OS=Homo sapiens GN=LUM PE=1 SV=2	LHINHNN#LTESVGPLPK	prediction
Q06033	Inter-alpha-trypsin inhibitor heavy chain H3 OS=Homo sapiens GN=ITIH3 PE=1 SV=2	NAHGEEKEN#LTAR	known
P02745	Complement C1q subcomponent subunit A OS=Homo sapiens GN=C1QA PE=1 SV=2	NPPMGGNVVIFDTVITNQEEPYQN#HSGR	known
P13671	Complement component C6 OS=Homo sapiens GN=C6 PE=1 SV=3	KLECNGEN#DCGDNSDER	unknown
P13671	Complement component C6 OS=Homo sapiens GN=C6 PE=1 SV=3	KLECNGEN#DCGDNSDERDCGR	unknown
P22792	Carboxypeptidase N subunit 2 OS=Homo sapiens GN=CPN2 PE=1 SV=3	LYLGSNN#LTALHPALFQN#LSK	known;known
P09871	Complement C1s subcomponent OS=Homo sapiens GN=C1S PE=1 SV=1	NCGVN#CSGDVFTALIGEIASPNYPKPYPENSR	known
P09871	Complement C1s subcomponent OS=Homo sapiens GN=C1S PE=1 SV=1	YTCEEPYYYMENGGGGEYHCAGN#GSWVNEVLGPELPK	known
P05160	Coagulation factor XIII B chain OS=Homo sapiens GN=F13B PE=1 SV=3	EHETCLAPELYNGN#YSTTQK	known
P05160	Coagulation factor XIII B chain OS=Homo sapiens GN=F13B PE=1 SV=3	KEHETCLAPELYNGN#YSTTQK	known
Q13201	Multimerin-1 OS=Homo sapiens GN=MMRN1 PE=1 SV=3	LQN#LTLPTN#ASIK	known;known
Q13201	Multimerin-1 OS=Homo sapiens GN=MMRN1 PE=1 SV=3	FNPGAESVVLSN#STLK	known
P40189	Interleukin-6 receptor subunit beta OS=Homo sapiens GN=IL6ST PE=1 SV=2	SHLQN#YTVN#ATK	known;known
P02760	Protein AMBP OS=Homo sapiens GN=AMBP PE=1 SV=1	YFYN#GTSMACETFQYGGCMGNGNNFVTEK	known
Q12913	Receptor-type tyrosine-protein phosphatase eta OS=Homo sapiens GN=PTPRJ PE=1 SV=3	IHVAGETDSSNLN#VSEPR	known
Q9GZK3	Olfactory receptor 2B2 OS=Homo sapiens GN=OR2B2 PE=2 SV=1	LHTPMYFFLSNLSLLDLCYTTSTVPQMLVN#ICNTR	unknown
P08603	Complement factor H OS=Homo sapiens GN=CFH PE=1 SV=4	WDPEVN#CSMAQIQLCPPPPQIPNSHN#MTTTLNYR	known;known
P10909	Clusterin OS=Homo sapiens GN=CLU PE=1 SV=1	QLEEFLN#QSSPFYFWMNGDR	known
P02745	Complement C1q subcomponent subunit A OS=Homo sapiens GN=C1QA PE=1 SV=2	RNPPMGGNVVIFDTVITNQEEPYQN#HSGR	known

Q9NU22	Midasin OS=Homo sapiens GN=MDN1 PE=1 SV=2	IAQFLESVANMFAAAQQLSQN#ISSETAQLLLVVSDGRGLFLEGK	unknown
Q9UHR4	Brain-specific angiogenesis inhibitor 1-associated protein 2-like protein 1 OS=Homo sapiens GN=BAIAP2L1 PE=1 SV=2	SISTVN#LSEN#SSVVIPPPDYLECLSMGAAADR	unknown;unknown
Q5XG99	LysM and putative peptidoglycan-binding domain-containing protein 4 OS=Homo sapiens GN=LYSMD4 PE=2 SV=2	IQASGETPNSLN#TTVIPN#GSMAMGTVPGQAPR	unknown;unknown
Q03518	Antigen peptide transporter 1 OS=Homo sapiens GN=TAP1 PE=1 SV=2	QETEFFQQN#QTGNIMSR	unknown
Q6ZRQ5	Protein MMS22-like OS=Homo sapiens GN=MMS22L PE=1 SV=3	QVVYGHQFMNLASDNLTN#ISLFEEHCETLLCDLISLSLNR	unknown
P01023	Alpha-2-macroglobulin OS=Homo sapiens GN=A2M PE=1 SV=3	GNEANYYSN#ATTDEHGLVQFSIN#TTNVMGTSLTVR	known;known
P01042	Kininogen-1 OS=Homo sapiens GN=KNG1 PE=1 SV=2	YNSQN#QSNNQFVLYR	known
P05155	Plasma protease C1 inhibitor OS=Homo sapiens GN=SERPING1 PE=1 SV=2	VLSN#NSDANLELINTWVAK	known
Q8WZ42	Titin OS=Homo sapiens GN=TTN PE=1 SV=4	VNFETTATSTILNIN#ECVR	unknown
Q9H8K7	Uncharacterized protein C10orf88 OS=Homo sapiens GN=C10orf88 PE=2 SV=2	VSDNSNIPNSELLPFLQN#LCSQVNHLHVGNK	unknown
Q9P266	Junctional protein associated with coronary artery disease OS=Homo sapiens GN=KIAA1462 PE=1 SV=3	SPPQNIPNPYLEDTVPIN#VCGGHSQQQSPTEK	unknown
Q9P2D1	Chromodomain-helicase-DNA-binding protein 7 OS=Homo sapiens GN=CHD7 PE=1 SV=3	YPYSNLNQGLVN#NTGMNQNLGLTNNTPMNQSVPR	unknown
P01009	Alpha-1-antitrypsin OS=Homo sapiens GN=SERPINA1 PE=1 SV=3	YLGN#ATAIFFLPDEGK	known
P0C0L4	Complement C4-A OS=Homo sapiens GN=C4A PE=1 SV=1	GLN#VTLSSTGR	known
P0C0L4	Complement C4-A OS=Homo sapiens GN=C4A PE=1 SV=1	N#TTCQDLQIEVTVK	known
P0C0L4	Complement C4-A OS=Homo sapiens GN=C4A PE=1 SV=1	FSDGLESN#SSTQFEVK	known
P0C0L5	Complement C4-B OS=Homo sapiens GN=C4B PE=1 SV=1	GLN#VTLSSTGR	known
P0C0L5	Complement C4-B OS=Homo sapiens GN=C4B PE=1 SV=1	N#TTCQDLQIEVTVK	known
P0C0L5	Complement C4-B OS=Homo sapiens GN=C4B PE=1 SV=1	FSDGLESN#SSTQFEVK	known

P01023	Alpha-2-macroglobulin OS=Homo sapiens GN=A2M PE=1 SV=3	VSN#QTLSLFFTVLQDVPVR	known
P01023	Alpha-2-macroglobulin OS=Homo sapiens GN=A2M PE=1 SV=3	GCVLLSYLN#ETVTVSASLESVR	known
P01023	Alpha-2-macroglobulin OS=Homo sapiens GN=A2M PE=1 SV=3	IITILEEEMN#VSVCGLYTYGKPVPGHVTVSICR	known
P01857	Ig gamma-1 chain C region OS=Homo sapiens GN=IGHG1 PE=1 SV=1	EEQYN#STYR	known
P01857	Ig gamma-1 chain C region OS=Homo sapiens GN=IGHG1 PE=1 SV=1	TKPREEQYN#STYR	known
P01859	Ig gamma-2 chain C region OS=Homo sapiens GN=IGHG2 PE=1 SV=2	EEQFN#STFR	known
P04114	Apolipoprotein B-100 OS=Homo sapiens GN=APOB PE=1 SV=2	YDFN#SSMLYSTAK	known
P04114	Apolipoprotein B-100 OS=Homo sapiens GN=APOB PE=1 SV=2	FVEGSHN#STVSLTTK	known
P04114	Apolipoprotein B-100 OS=Homo sapiens GN=APOB PE=1 SV=2	FEVDSPVYN#ATWSASLK	known
P04114	Apolipoprotein B-100 OS=Homo sapiens GN=APOB PE=1 SV=2	QVLFLDTVYGN#CSTHFTVK	known
P04114	Apolipoprotein B-100 OS=Homo sapiens GN=APOB PE=1 SV=2	IQSPLFTLDANADIGN#GTTSANEAGIAASITAK	known
P04114	Apolipoprotein B-100 OS=Homo sapiens GN=APOB PE=1 SV=2	QVFPGLNYCTSGAYSN#ASSTDSASYYPLTGDTR	prediction
P01011	Alpha-1-antichymotrypsin OS=Homo sapiens GN=SERPINA3 PE=1 SV=2	YTGN#ASALFILPDQDK	known
P01011	Alpha-1-antichymotrypsin OS=Homo sapiens GN=SERPINA3 PE=1 SV=2	FN#LTETSEAEIHQSFQHLLR	known
P00738	Haptoglobin OS=Homo sapiens GN=HP PE=1 SV=1	NLFLN#HSENATAK	known
P00738	Haptoglobin OS=Homo sapiens GN=HP PE=1 SV=1	VVLHPN#YSQVDIGLIK	known
P01871	Ig mu chain C region OS=Homo sapiens GN=IGHM PE=1 SV=3	GLTFQQN#ASSMCVPDQDTAIR	known
P01871	Ig mu chain C region OS=Homo sapiens GN=IGHM PE=1 SV=3	STGKPTLYN#VSLVMSDTAGTCY	known
P01871	Ig mu chain C region OS=Homo sapiens GN=IGHM PE=1 SV=3	THTN#ISESHPNATFSAVGEASICEDDWNSGER	known

P02763	Alpha-1-acid glycoprotein 1 OS=Homo sapiens GN=ORM1 PE=1 SV=1	EN#GTISR	known
P02763	Alpha-1-acid glycoprotein 1 OS=Homo sapiens GN=ORM1 PE=1 SV=1	SVQEIQATFFYFTPN#K	known
P02763	Alpha-1-acid glycoprotein 1 OS=Homo sapiens GN=ORM1 PE=1 SV=1	SVQEIQATFFYFTPN#KTEDTIFLR	known
P02766	Transthyretin OS=Homo sapiens GN=TTR PE=1 SV=1	ALGISPFHEHAEVVFTAN#DSGPR	known
P02787	Serotransferrin OS=Homo sapiens GN=TF PE=1 SV=3	QQQHLFGSN#VTDCSGNFCLFR	known
P01860	Ig gamma-3 chain C region OS=Homo sapiens GN=IGHG3 PE=1 SV=2	EEQYN#STFR	known
P01008	Antithrombin-III OS=Homo sapiens GN=SERPINC1 PE=1 SV=1	LGACN#DTLQQLMEVFK	known
P01008	Antithrombin-III OS=Homo sapiens GN=SERPINC1 PE=1 SV=1	SLTFN#ETYQDISELVYGAK	known
P00450	Ceruloplasmin OS=Homo sapiens GN=CP PE=1 SV=1	AGLQAFFQVQECN#K	known
P00450	Ceruloplasmin OS=Homo sapiens GN=CP PE=1 SV=1	EHEGAIYPDN#TTDFQR	known
P00450	Ceruloplasmin OS=Homo sapiens GN=CP PE=1 SV=1	EN#LTAPGSDSAVFFEQGTTR	known
P08603	Complement factor H OS=Homo sapiens GN=CFH PE=1 SV=4	SPDVIN#GSPISQK	known
P08603	Complement factor H OS=Homo sapiens GN=CFH PE=1 SV=4	ISEEN#ETTCYMGK	known
P08603	Complement factor H OS=Homo sapiens GN=CFH PE=1 SV=4	IPCSQPPQIEHGTIN#SSR	known
P01876	Ig alpha-1 chain C region OS=Homo sapiens GN=IGHA1 PE=1 SV=2	LAGKPTHVN#VSVVMAEVDGTCY	known
P01876	Ig alpha-1 chain C region OS=Homo sapiens GN=IGHA1 PE=1 SV=2	LSLHRPALEDLLLGSEAN#LTCTLTGLR	known
P02790	Hemopexin OS=Homo sapiens GN=HPX PE=1 SV=2	SWPAVGN#CSSALR	known
P02790	Hemopexin OS=Homo sapiens GN=HPX PE=1 SV=2	ALPQPQN#VTSLLGCTH	known
P02790	Hemopexin OS=Homo sapiens GN=HPX PE=1 SV=2	CSDGWSFDATTLDDN#GTMLFFK	known

P01861	Ig gamma-4 chain C region OS=Homo sapiens GN=IGHG4 PE=1 SV=1	EEQFN#STYR	known
P19652	Alpha-1-acid glycoprotein 2 OS=Homo sapiens GN=ORM2 PE=1 SV=2	SVQEIQATFFYFTPN#K	known
P19652	Alpha-1-acid glycoprotein 2 OS=Homo sapiens GN=ORM2 PE=1 SV=2	SVQEIQATFFYFTPN#KTEDTIFLR	known
P02765	Alpha-2-HS-glycoprotein OS=Homo sapiens GN=AHSG PE=1 SV=1	VCQDCPLLAPLN#DTR	known
P02765	Alpha-2-HS-glycoprotein OS=Homo sapiens GN=AHSG PE=1 SV=1	KVCQDCPLLAPLN#DTR	known
P01042	Kininogen-1 OS=Homo sapiens GN=KNG1 PE=1 SV=2	ITYSIVQTN#CSK	known
P01042	Kininogen-1 OS=Homo sapiens GN=KNG1 PE=1 SV=2	KYNSQN#QSNNQFVLYR	known
P05090	Apolipoprotein D OS=Homo sapiens GN=APOD PE=1 SV=1	ADGTVNQIEGEATPVN#LTEPAKLEVK	known
P25311	Zinc-alpha-2-glycoprotein OS=Homo sapiens GN=AZGP1 PE=1 SV=2	DIVEYYNDSN#GSHVLQGR	known
P05155	Plasma protease C1 inhibitor OS=Homo sapiens GN=SERPING1 PE=1 SV=2	DTFVN#ASR	known
P05155	Plasma protease C1 inhibitor OS=Homo sapiens GN=SERPING1 PE=1 SV=2	ASSNPN#ATSSSSQDPESLQDR	known
P05155	Plasma protease C1 inhibitor OS=Homo sapiens GN=SERPING1 PE=1 SV=2	VGQLQLSHN#LSLVILVPQNLK	known
P04220	Ig mu heavy chain disease protein OS=Homo sapiens PE=1 SV=1	STGKPTLYN#VSLVMSDTAGTCY	unknown
P04220	Ig mu heavy chain disease protein OS=Homo sapiens PE=1 SV=1	THTN#ISESHPNATFSAVGEASICEDDWDSGER	unknown
Q08380	Galectin-3-binding protein OS=Homo sapiens GN=LGALS3BP PE=1 SV=1	DAGVVCTN#ETR	known
Q08380	Galectin-3-binding protein OS=Homo sapiens GN=LGALS3BP PE=1 SV=1	AAIPSALDTN#SSK	known
Q08380	Galectin-3-binding protein OS=Homo sapiens GN=LGALS3BP PE=1 SV=1	ALGFEN#ATQALGR	known
Q08380	Galectin-3-binding protein OS=Homo sapiens GN=LGALS3BP PE=1 SV=1	GLN#LTEDTYKPR	known
Q08380	Galectin-3-binding protein OS=Homo sapiens GN=LGALS3BP PE=1 SV=1	TVIRPFYLTN#SSGVD	known

Q08380	Galectin-3-binding protein OS=Homo sapiens GN=LGALS3BP PE=1 SV=1	EPGSN#VTMSVDAECVPMVR	known
P02749	Beta-2-glycoprotein 1 OS=Homo sapiens GN=APOH PE=1 SV=3	LGN#WSAMPSCK	known
P19823	Inter-alpha-trypsin inhibitor heavy chain H2 OS=Homo sapiens GN=ITIH2 PE=1 SV=2	GAFISN#FSMTVDGK	known
P01877	Ig alpha-2 chain C region OS=Homo sapiens GN=IGHA2 PE=1 SV=3	TPLTAN#ITK	known
P01877	Ig alpha-2 chain C region OS=Homo sapiens GN=IGHA2 PE=1 SV=3	LSLHRPALEDLLLGSEAN#LTCTLTGLR	known
P01877	Ig alpha-2 chain C region OS=Homo sapiens GN=IGHA2 PE=1 SV=3	VFPLSLDSTPQDGNVVVACLVQGFFPQEPLSVTWSESGQN#VT AR	known
P04004	Vitronectin OS=Homo sapiens GN=VTN PE=1 SV=1	N#GSLFAFR	known
Q14624	Inter-alpha-trypsin inhibitor heavy chain H4 OS=Homo sapiens GN=ITIH4 PE=1 SV=4	AFITN#FSMIIDGMTYPGIIK	known
Q14624	Inter-alpha-trypsin inhibitor heavy chain H4 OS=Homo sapiens GN=ITIH4 PE=1 SV=4	LPTQN#ITFQTESSVAEQEAEFQSPK	known
P07357	Complement component C8 alpha chain OS=Homo sapiens GN=C8A PE=1 SV=2	GGSSGWSGGLAQN#R	known
P00751	Complement factor B OS=Homo sapiens GN=CFB PE=1 SV=2	ALQAVYSMMSWPDDVPPEGWN#R	known
P00751	Complement factor B OS=Homo sapiens GN=CFB PE=1 SV=2	SPYYN#VSDEISFHCYDGYTLR	known
P01591	Immunoglobulin J chain OS=Homo sapiens GN=IGJ PE=1 SV=4	EN#ISDPTSPLR	known
P01591	Immunoglobulin J chain OS=Homo sapiens GN=IGJ PE=1 SV=4	IIVPLNNREN#ISDPTSPLR	known
P01591	Immunoglobulin J chain OS=Homo sapiens GN=IGJ PE=1 SV=4	CDPTEVELDNQIVTATQSN#ICDEDSATETCYTYDR	unknown
P08185	Corticosteroid-binding globulin OS=Homo sapiens GN=SERPINA6 PE=1 SV=1	AQLLQGLGFN#LTER	known
P10909	Clusterin OS=Homo sapiens GN=CLU PE=1 SV=1	HN#STGCLR	known
P10909	Clusterin OS=Homo sapiens GN=CLU PE=1 SV=1	EDALN#ETR	known
P10909	Clusterin OS=Homo sapiens GN=CLU PE=1 SV=1	KKEDALN#ETR	known

P10909	Clusterin OS=Homo sapiens GN=CLU PE=1 SV=1	LAN#LTQGEDQYYLR	known
P10909	Clusterin OS=Homo sapiens GN=CLU PE=1 SV=1	ELPGVCN#ETMMALWEECKPCLK	known
P10909	Clusterin OS=Homo sapiens GN=CLU PE=1 SV=1	LKELPGVCN#ETMMALWEECKPCLK	known
O75882	Attractin OS=Homo sapiens GN=ATRN PE=1 SV=2	EWLPLN#R	known
O75882	Attractin OS=Homo sapiens GN=ATRN PE=1 SV=2	GICN#SSDVR	known
O75882	Attractin OS=Homo sapiens GN=ATRN PE=1 SV=2	CIN#QSICEK	known
O75882	Attractin OS=Homo sapiens GN=ATRN PE=1 SV=2	DLDMFIN#ASK	known
O75882	Attractin OS=Homo sapiens GN=ATRN PE=1 SV=2	N#HSCSEGQISIFR	known
O75882	Attractin OS=Homo sapiens GN=ATRN PE=1 SV=2	ISN#SSDTVECECSENWK	known
O75882	Attractin OS=Homo sapiens GN=ATRN PE=1 SV=2	MPSQAPTGNFYPQPLLN#SSMCLEDSR	known
O75882	Attractin OS=Homo sapiens GN=ATRN PE=1 SV=2	GCSCFSDWQGPGCSVPVPAN#QSFWTR	prediction
P04275	von Willebrand factor OS=Homo sapiens GN=VWF PE=1 SV=4	IGEADFN#R	known
P04275	von Willebrand factor OS=Homo sapiens GN=VWF PE=1 SV=4	MEACMLN#GTVIGPGK	prediction
P04275	von Willebrand factor OS=Homo sapiens GN=VWF PE=1 SV=4	ASPPSSSCN#ISSGEMQK	prediction
P04275	von Willebrand factor OS=Homo sapiens GN=VWF PE=1 SV=4	N#VSCPQLEVPVCPSGFQLSCK	known
P04275	von Willebrand factor OS=Homo sapiens GN=VWF PE=1 SV=4	GLQPTLTNPGECRPN#FTCACR	prediction
P04275	von Willebrand factor OS=Homo sapiens GN=VWF PE=1 SV=4	TTCNPCPLGYKEEN#NTGECCGR	unknown
P04275	von Willebrand factor OS=Homo sapiens GN=VWF PE=1 SV=4	HCDGN#VSSCGDHPSEGCFCPPDK	prediction
P20851	C4b-binding protein beta chain OS=Homo sapiens GN=C4BPB PE=1 SV=1	TLFCN#ASK	known

P20851	C4b-binding protein beta chain OS=Homo sapiens GN=C4BPB PE=1 SV=1	EWDN#TTTECR	known
P04003	C4b-binding protein alpha chain OS=Homo sapiens GN=C4BPA PE=1 SV=2	SRPAN#HCVYFYGDEISFSCHETSR	unknown
P04003	C4b-binding protein alpha chain OS=Homo sapiens GN=C4BPA PE=1 SV=2	FSLLGHASISCTVEN#ETIGVWRPSPPTCEK	known
P04003	C4b-binding protein alpha chain OS=Homo sapiens GN=C4BPA PE=1 SV=2	LSVDKDQYVEPEN#VTIQCDSGYGVVGPQSITCSGNR	known
P04196	Histidine-rich glycoprotein OS=Homo sapiens GN=HRG PE=1 SV=1	HSHNN#NSSDLHPHK	known
P04196	Histidine-rich glycoprotein OS=Homo sapiens GN=HRG PE=1 SV=1	VEN#TTVYYLVLDVQESDCSVLSR	known
P04196	Histidine-rich glycoprotein OS=Homo sapiens GN=HRG PE=1 SV=1	IADAHLDRVEN#TTVYYLVLDVQESDCSVLSR	known
P05546	Heparin cofactor 2 OS=Homo sapiens GN=SERPIND1 PE=1 SV=3	DFVN#ASSK	known
P05546	Heparin cofactor 2 OS=Homo sapiens GN=SERPIND1 PE=1 SV=3	N#LSMPLLPADFHK	known
P06681	Complement C2 OS=Homo sapiens GN=C2 PE=1 SV=2	TMFPN#LTDVR	known
P06681	Complement C2 OS=Homo sapiens GN=C2 PE=1 SV=2	QSVPAHFVALN#GSK	known
P06681	Complement C2 OS=Homo sapiens GN=C2 PE=1 SV=2	LTDTICGVGNMSAN#ASDQER	known
P10643	Complement component C7 OS=Homo sapiens GN=C7 PE=1 SV=2	N#YTLTGR	known
P10643	Complement component C7 OS=Homo sapiens GN=C7 PE=1 SV=2	INNDFNYEFYN#STWSYVK	known
P02750	Leucine-rich alpha-2-glycoprotein OS=Homo sapiens GN=LRG1 PE=1 SV=2	MFSQN#DTR	known
P02750	Leucine-rich alpha-2-glycoprotein OS=Homo sapiens GN=LRG1 PE=1 SV=2	KLPPGLLAN#FTLLR	known
P43251	Biotinidase OS=Homo sapiens GN=BTD PE=1 SV=2	FN#DTEVLQR	known
P43251	Biotinidase OS=Homo sapiens GN=BTD PE=1 SV=2	NPVGLIGAEN#ATGETDPSHSK	known
P43251	Biotinidase OS=Homo sapiens GN=BTD PE=1 SV=2	DVQIIVFPEDGIHGFN#FTR	known

P43251	Biotinidase OS=Homo sapiens GN=BTD PE=1 SV=2	WNPCLEPHRFN#DTEVLQR	known
P43251	Biotinidase OS=Homo sapiens GN=BTD PE=1 SV=2	WNVNAPPTFHSEMMYDN#FTLVPVWGK	known
P04217	Alpha-1B-glycoprotein OS=Homo sapiens GN=A1BG PE=1 SV=4	EGDHEFLEVPEAQEDVEATFPVHQPGN#YSCSYR	known
P04217	Alpha-1B-glycoprotein OS=Homo sapiens GN=A1BG PE=1 SV=4	REGDHEFLEVPEAQEDVEATFPVHQPGN#YSCSYR	known
P04217	Alpha-1B-glycoprotein OS=Homo sapiens GN=A1BG PE=1 SV=4	FQSPAGTEALFELHNISVADSAN#YSCVYVDLKPPFGGSAPSER	known
P02748	Complement component C9 OS=Homo sapiens GN=C9 PE=1 SV=2	N#ETYQLFLSYSSK	known
P02748	Complement component C9 OS=Homo sapiens GN=C9 PE=1 SV=2	AVN#ITSENLIDDVVSLIR	known
P05543	Thyroxine-binding globulin OS=Homo sapiens GN=SERPINA7 PE=1 SV=2	VTACHSSQPN#ATLYK	known
P05543	Thyroxine-binding globulin OS=Homo sapiens GN=SERPINA7 PE=1 SV=2	TLYETEVFSTDFSN#ISAAK	known
P55058	Phospholipid transfer protein OS=Homo sapiens GN=PLTP PE=1 SV=1	N#WSLPNR	known
P55058	Phospholipid transfer protein OS=Homo sapiens GN=PLTP PE=1 SV=1	VSN#VSCQASVSR	known
P55058	Phospholipid transfer protein OS=Homo sapiens GN=PLTP PE=1 SV=1	EGHFYYN#ISEVK	known
P55058	Phospholipid transfer protein OS=Homo sapiens GN=PLTP PE=1 SV=1	IYSN#HSALESLALIPLQAPLK	known
P00734	Prothrombin OS=Homo sapiens GN=F2 PE=1 SV=2	GHVN#ITR	known
P00734	Prothrombin OS=Homo sapiens GN=F2 PE=1 SV=2	YPHKPEIN#STTHPGADLQENFCR	known
P00734	Prothrombin OS=Homo sapiens GN=F2 PE=1 SV=2	WVLTAAHCLLYPPWDKN#FTENDLLVR	known
P05156	Complement factor I OS=Homo sapiens GN=CFI PE=1 SV=2	LISN#CSK	prediction
P05156	Complement factor I OS=Homo sapiens GN=CFI PE=1 SV=2	N#GTAVCATNR	known
P05156	Complement factor I OS=Homo sapiens GN=CFI PE=1 SV=2	LSDLSIN#STECLHVHCR	known

P05156	Complement factor I OS=Homo sapiens GN=CFI PE=1 SV=2	ACDGIN#DCGDQSDELCCK	unknown
P02751	Fibronectin OS=Homo sapiens GN=FN1 PE=1 SV=4	HEEGHMLN#CTCFGQGR	known
P02751	Fibronectin OS=Homo sapiens GN=FN1 PE=1 SV=4	DQCIVDDITYNVN#DTFHK	known
P01616	Ig kappa chain V-II region MIL OS=Homo sapiens PE=1 SV=1	FSGSGSGTN#FTLK	unknown
P43652	Afamin OS=Homo sapiens GN=AFM PE=1 SV=1	HN#FSHCCSK	known
P43652	Afamin OS=Homo sapiens GN=AFM PE=1 SV=1	YAEDKFN#ETTEK	known
P49908	Selenoprotein P OS=Homo sapiens GN=SEPP1 PE=1 SV=3	CGN#CSLTTLK	By similarity
P49908	Selenoprotein P OS=Homo sapiens GN=SEPP1 PE=1 SV=3	CGN#CSLTTLKDEDFCK	By similarity
P49908	Selenoprotein P OS=Homo sapiens GN=SEPP1 PE=1 SV=3	VSEHIPVYQQEEN#QTDVWTLLNGSK	known
O95497	Pantetheinase OS=Homo sapiens GN=VNN1 PE=1 SV=2	MTGSGIYAPN#SSR	known
O95497	Pantetheinase OS=Homo sapiens GN=VNN1 PE=1 SV=2	LTGVAGN#YTVCQK	known
O95497	Pantetheinase OS=Homo sapiens GN=VNN1 PE=1 SV=2	LLLSQLDSHPSHSAVVN#WTSYASSIEALSSGNK	known
P27169	Serum paraoxonase/arylesterase 1 OS=Homo sapiens GN=PON1 PE=1 SV=3	HAN#WTLTPLK	known
P27169	Serum paraoxonase/arylesterase 1 OS=Homo sapiens GN=PON1 PE=1 SV=3	VTQVYAEN#GTVLQGSTVASVYK	known
P01833	Polymeric immunoglobulin receptor OS=Homo sapiens GN=PIGR PE=1 SV=4	VPGN#VTAVLGETLK	known
P01833	Polymeric immunoglobulin receptor OS=Homo sapiens GN=PIGR PE=1 SV=4	WN#NTGCQALPSQDEGPSK	known
P03952	Plasma kallikrein OS=Homo sapiens GN=KLKB1 PE=1 SV=1	GVNFN#VSK	known
P03952	Plasma kallikrein OS=Homo sapiens GN=KLKB1 PE=1 SV=1	GVN#VCQETCTK	unknown
P03952	Plasma kallikrein OS=Homo sapiens GN=KLKB1 PE=1 SV=1	IYSGILN#LSDITK	known

P03952	Plasma kallikrein OS=Homo sapiens GN=KLKB1 PE=1 SV=1	IYPGVDFGGEELN#VTFVK	known
P03952	Plasma kallikrein OS=Homo sapiens GN=KLKB1 PE=1 SV=1	LQAPLN#YTEFQKPICLPSK	known
P03952	Plasma kallikrein OS=Homo sapiens GN=KLKB1 PE=1 SV=1	IVGGTN#SSWGEWPWQVSLQVK	known
P29622	Kallistatin OS=Homo sapiens GN=SERPINA4 PE=1 SV=3	DFYVDEN#TTVR	known
P29622	Kallistatin OS=Homo sapiens GN=SERPINA4 PE=1 SV=3	FLN#DTMAVYEAK	known
P29622	Kallistatin OS=Homo sapiens GN=SERPINA4 PE=1 SV=3	SQILEGLGFN#LTELSESDVHR	known
P07996	Thrombospondin-1 OS=Homo sapiens GN=THBS1 PE=1 SV=2	VVN#STTGPGEHLR	known
P07996	Thrombospondin-1 OS=Homo sapiens GN=THBS1 PE=1 SV=2	VSCPIMPCSN#ATVPDGECCPR	prediction
P19827	Inter-alpha-trypsin inhibitor heavy chain H1 OS=Homo sapiens GN=ITIH1 PE=1 SV=3	ICDLLVANNHFAHFFAPQN#LTNMNK	known
P51884	Lumican OS=Homo sapiens GN=LUM PE=1 SV=2	KLHINHNN#LTESVGPLPK	prediction
P51884	Lumican OS=Homo sapiens GN=LUM PE=1 SV=2	LGSFEGLVN#LTFIHLQHNR	known
P51884	Lumican OS=Homo sapiens GN=LUM PE=1 SV=2	AFEN#VTDLQWLILDHNLLENSK	known
O95445	Apolipoprotein M OS=Homo sapiens GN=APOM PE=1 SV=2	TELFSSSCPGGIMLN#ETGQGYQR	known
Q9Y6R7	IgGFc-binding protein OS=Homo sapiens GN=FCGBP PE=1 SV=3	N#ECGILADPK	unknown
Q9Y6R7	IgGFc-binding protein OS=Homo sapiens GN=FCGBP PE=1 SV=3	VITVQVAN#FTLR	known
Q9Y6R7	IgGFc-binding protein OS=Homo sapiens GN=FCGBP PE=1 SV=3	SVTLQIYN#HSLTLSAR	known
Q9Y6R7	IgGFc-binding protein OS=Homo sapiens GN=FCGBP PE=1 SV=3	YLPVN#SSLLTSDCSER	unknown
P04278	Sex hormone-binding globulin OS=Homo sapiens GN=SHBG PE=1 SV=2	LDVDQALN#R	known
P04278	Sex hormone-binding globulin OS=Homo sapiens GN=SHBG PE=1 SV=2	SHEIWTHSCPQSPGN#GTDASH	known

Q8TF66	Leucine-rich repeat-containing protein 15 OS=Homo sapiens GN=LRRC15 PE=1 SV=2	MLANLQN#ISLQNNR	prediction
P80108	Phosphatidylinositol-glycan-specific phospholipase D OS=Homo sapiens GN=GPLD1 PE=1 SV=3	LNVEAAN#WTVR	prediction
P80108	Phosphatidylinositol-glycan-specific phospholipase D OS=Homo sapiens GN=GPLD1 PE=1 SV=3	N#LTTSLTESVDR	prediction
P80108	Phosphatidylinositol-glycan-specific phospholipase D OS=Homo sapiens GN=GPLD1 PE=1 SV=3	LGTSLSSGHVLMN#GTLK	known
Q06033	Inter-alpha-trypsin inhibitor heavy chain H3 OS=Homo sapiens GN=ITIH3 PE=1 SV=2	KNAHGEEKEN#LTAR	known
P05154	Plasma serine protease inhibitor OS=Homo sapiens GN=SERPINA5 PE=1 SV=3	VVGVPYQGN#ATALFILPSEGK	known
P01596	Ig kappa chain V-I region CAR OS=Homo sapiens PE=1 SV=1	ASQN#ISSWLAWYQQKPGK	known
P00748	Coagulation factor XII OS=Homo sapiens GN=F12 PE=1 SV=3	N#HSCEPCQTLAVR	known
P00748	Coagulation factor XII OS=Homo sapiens GN=F12 PE=1 SV=3	RN#HSCEPCQTLAVR	known
P23142	Fibulin-1 OS=Homo sapiens GN=FBLN1 PE=1 SV=4	CATPHGDN#ASLEATFVK	known
Q9BY67	Cell adhesion molecule 1 OS=Homo sapiens GN=CADM1 PE=1 SV=2	VSLTN#VSISDEGR	known
Q9BY67	Cell adhesion molecule 1 OS=Homo sapiens GN=CADM1 PE=1 SV=2	FQLLN#FSSSELK	known
P13671	Complement component C6 OS=Homo sapiens GN=C6 PE=1 SV=3	VLN#FTTK	known
Q86VB7	Scavenger receptor cysteine-rich type 1 protein M130 OS=Homo sapiens GN=CD163 PE=1 SV=2	GN#ESALWDCK	unknown
Q86VB7	Scavenger receptor cysteine-rich type 1 protein M130 OS=Homo sapiens GN=CD163 PE=1 SV=2	EDAAVN#CTDISVQK	known
Q86VB7	Scavenger receptor cysteine-rich type 1 protein M130 OS=Homo sapiens GN=CD163 PE=1 SV=2	HSN#CTHQQDAGVTCSDGSNLEMR	known
Q9UGM5	Fetuin-B OS=Homo sapiens GN=FETUB PE=1 SV=2	VLYLAAYN#CTLRPVSK	known
P22792	Carboxypeptidase N subunit 2 OS=Homo sapiens GN=CPN2 PE=1 SV=3	AFGSNPN#LTK	prediction
P05362	Intercellular adhesion molecule 1 OS=Homo sapiens GN=ICAM1 PE=1 SV=2	AN#LTVVLLR	known

P05362	Intercellular adhesion molecule 1 OS=Homo sapiens GN=ICAM1 PE=1 SV=2	DHHGAN#FSCR	known
P05362	Intercellular adhesion molecule 1 OS=Homo sapiens GN=ICAM1 PE=1 SV=2	LNPTVTYGN#DSFSAK	known
P05160	Coagulation factor XIII B chain OS=Homo sapiens GN=F13B PE=1 SV=3	HGVIISSTVDTYEN#GSSVEYR	known
Q9UK55	Protein Z-dependent protease inhibitor OS=Homo sapiens GN=SERPINA10 PE=1 SV=1	ETFFN#LSK	known
P17936	Insulin-like growth factor-binding protein 3 OS=Homo sapiens GN=IGFBP3 PE=1 SV=2	GLCVN#ASAVSR	known
Q96PD5	N-acetylmuramoyl-L-alanine amidase OS=Homo sapiens GN=PGLYRP2 PE=1 SV=1	GFGVAIVGN#YTAALPTEAALR	known
Q13201	Multimerin-1 OS=Homo sapiens GN=MMRN1 PE=1 SV=3	HPFTGDN#CTIK	prediction
Q13201	Multimerin-1 OS=Homo sapiens GN=MMRN1 PE=1 SV=3	TQAALSN#LTCCIDR	prediction
Q9ULI3	Protein HEG homolog 1 OS=Homo sapiens GN=HEG1 PE=1 SV=3	N#SSGPDLSWLHFYR	unknown
P13473	Lysosome-associated membrane glycoprotein 2 OS=Homo sapiens GN=LAMP2 PE=1 SV=2	WQMN#FTVR	known
P13473	Lysosome-associated membrane glycoprotein 2 OS=Homo sapiens GN=LAMP2 PE=1 SV=2	VQPFN#VTQGK	known
P13473	Lysosome-associated membrane glycoprotein 2 OS=Homo sapiens GN=LAMP2 PE=1 SV=2	VASVININPN#TTHSTGSCR	known
P04070	Vitamin K-dependent protein C OS=Homo sapiens GN=PROC PE=1 SV=1	EVFVHPN#YSK	known
P04066	Tissue alpha-L-fucosidase OS=Homo sapiens GN=FUCA1 PE=1 SV=4	N#TTSVWYTSK	known
P55285	Cadherin-6 OS=Homo sapiens GN=CDH6 PE=1 SV=1	IFNIDSGN#GSIFTSK	prediction
P55285	Cadherin-6 OS=Homo sapiens GN=CDH6 PE=1 SV=1	EDAQIN#TTIGSVTAQDPDAAR	known
P40189	Interleukin-6 receptor subunit beta OS=Homo sapiens GN=IL6ST PE=1 SV=2	EQYTIIN#R	known
P40189	Interleukin-6 receptor subunit beta OS=Homo sapiens GN=IL6ST PE=1 SV=2	LTVN#LTNDR	known
P40189	Interleukin-6 receptor subunit beta OS=Homo sapiens GN=IL6ST PE=1 SV=2	N#LSCIVNEGK	known

Q12866	Tyrosine-protein kinase Mer OS=Homo sapiens GN=MERTK PE=1 SV=2	ELLEEVGQN#GSR	known
P55056	Apolipoprotein C-IV OS=Homo sapiens GN=APOC4 PE=1 SV=1	ELLETVVN#R	known
P01033	Metalloproteinase inhibitor 1 OS=Homo sapiens GN=TIMP1 PE=1 SV=1	FVGTPEVN#QTTLYQR	known
Q9Y5Y7	Lymphatic vessel endothelial hyaluronic acid receptor 1 OS=Homo sapiens GN=LYVE1 PE=1 SV=2	ANQQLN#FTEAK	known
Q9Y5Y7	Lymphatic vessel endothelial hyaluronic acid receptor 1 OS=Homo sapiens GN=LYVE1 PE=1 SV=2	KANQQLN#FTEAK	known
P14151	L-selectin OS=Homo sapiens GN=SELL PE=1 SV=2	DN#YTDLVAIQNK	known
P26927	Hepatocyte growth factor-like protein OS=Homo sapiens GN=MST1 PE=1 SV=2	GTAN#TTTAGVPCQR	known
Q07954	Prolow-density lipoprotein receptor-related protein 1 OS=Homo sapiens GN=LRP1 PE=1 SV=2	IETILLN#GTDR	known
Q07954	Prolow-density lipoprotein receptor-related protein 1 OS=Homo sapiens GN=LRP1 PE=1 SV=2	LN#GTDPIVAADSK	known
P03951	Coagulation factor XI OS=Homo sapiens GN=F11 PE=1 SV=1	GINYN#SSVAK	known
P03951	Coagulation factor XI OS=Homo sapiens GN=F11 PE=1 SV=1	VYSGILN#QSEIK	known
P15144	Aminopeptidase N OS=Homo sapiens GN=ANPEP PE=1 SV=4	N#ATLVNEADKLR	known
P15144	Aminopeptidase N OS=Homo sapiens GN=ANPEP PE=1 SV=4	GPSTPLPEDPNWN#VTEFHTTPK	known
P06276	Cholinesterase OS=Homo sapiens GN=BCHE PE=1 SV=1	EN#ETEIIK	known
P06276	Cholinesterase OS=Homo sapiens GN=BCHE PE=1 SV=1	WSDIWN#ATK	known
P33151	Cadherin-5 OS=Homo sapiens GN=CDH5 PE=1 SV=5	EVYPWYN#LTVEAK	known
P48357	Leptin receptor OS=Homo sapiens GN=LEPR PE=1 SV=2	YSEN#STTVIR	known
P36955	Pigment epithelium-derived factor OS=Homo sapiens GN=SERPINF1 PE=1 SV=4	VTQN#LTLIEESLTSEFIHDIDR	known
P07339	Cathepsin D OS=Homo sapiens GN=CTSD PE=1 SV=1	GSLSYLN#VTR	known

O00391	Sulfhydryl oxidase 1 OS=Homo sapiens GN=QSOX1 PE=1 SV=3	N#GSGAVFPVAGADVQTLR	known
Q15063	Periostin OS=Homo sapiens GN=POSTN PE=1 SV=2	EVN#DTLLVNELK	known
P02671	Fibrinogen alpha chain OS=Homo sapiens GN=FGA PE=1 SV=2	MDGSLNFN#R	known
Q96IY4	Carboxypeptidase B2 OS=Homo sapiens GN=CPB2 PE=1 SV=2	QVHFFVN#ASDVDNVK	known
P04180	Phosphatidylcholine-sterol acyltransferase OS=Homo sapiens GN=LCAT PE=1 SV=1	MAWPEDHVFISTPSFN#YTGR	known
P04180	Phosphatidylcholine-sterol acyltransferase OS=Homo sapiens GN=LCAT PE=1 SV=1	AELSN#HTRPVILVPGCLGNQLEAK	known
Q9H8L6	Multimerin-2 OS=Homo sapiens GN=MMRN2 PE=1 SV=2	FN#TTYINIGSSYFPEHGYFR	known
P13598	Intercellular adhesion molecule 2 OS=Homo sapiens GN=ICAM2 PE=1 SV=2	N#FSCLAVLDLMSR	known
P13598	Intercellular adhesion molecule 2 OS=Homo sapiens GN=ICAM2 PE=1 SV=2	AAPAPQEATATFN#STADR	known
P15151	Poliovirus receptor OS=Homo sapiens GN=PVR PE=1 SV=2	VEDEGN#YTCLFVTFPQGSR	known
P32942	Intercellular adhesion molecule 3 OS=Homo sapiens GN=ICAM3 PE=1 SV=2	EIVCN#VTLGGER	known
Q6EMK4	Vasorin OS=Homo sapiens GN=VASN PE=1 SV=1	LHEITN#ETFR	known
P13688	Carcinoembryonic antigen-related cell adhesion molecule 1 OS=Homo sapiens GN=CEACAM1 PE=1 SV=2	LSQGN#TTLSINPVK	known
Q14126	Desmoglein-2 OS=Homo sapiens GN=DSG2 PE=1 SV=2	YVQN#GTYTVK	known
Q14126	Desmoglein-2 OS=Homo sapiens GN=DSG2 PE=1 SV=2	IN#ATDADEPNTLNSK	known
Q15223	Poliovirus receptor-related protein 1 OS=Homo sapiens GN=PVRL1 PE=1 SV=3	NPN#GTVTVISR	known
P35442	Thrombospondin-2 OS=Homo sapiens GN=THBS2 PE=1 SV=2	VVN#STTGTGEHLR	known
P12259	Coagulation factor V OS=Homo sapiens GN=F5 PE=1 SV=4	TWN#QSIALR	prediction
P12259	Coagulation factor V OS=Homo sapiens GN=F5 PE=1 SV=4	DPPSDLLLLKQSN#SSK	prediction

P53634	Dipeptidyl peptidase 1 OS=Homo sapiens GN=CTSC PE=1 SV=2	DVN#CSVMGPQEK	known
Q86SQ4	G-protein coupled receptor 126 OS=Homo sapiens GN=GPR126 PE=1 SV=3	ILSN#LSCNVK	known
Q02413	Desmoglein-1 OS=Homo sapiens GN=DSG1 PE=1 SV=2	TGEIN#ITSIVDR	known
P02679	Fibrinogen gamma chain OS=Homo sapiens GN=FGG PE=1 SV=3	VDKDLQSLEDILHQVEN#K	known
Q99784	Noelin OS=Homo sapiens GN=OLFM1 PE=1 SV=4	VQN#MSQSIEVLDR	known
Q99784	Noelin OS=Homo sapiens GN=OLFM1 PE=1 SV=4	SMVDFMNTDN#FTSHR	known
Q99784	Noelin OS=Homo sapiens GN=OLFM1 PE=1 SV=4	LDPVSLQTLQTWN#TSYPK	known
Q9BTY2	Plasma alpha-L-fucosidase OS=Homo sapiens GN=FUCA2 PE=1 SV=2	SQN#DTVTPDVWYTSKPK	prediction
Q04756	Hepatocyte growth factor activator OS=Homo sapiens GN=HGFAC PE=1 SV=1	CFLGN#GTGYR	prediction
Q04756	Hepatocyte growth factor activator OS=Homo sapiens GN=HGFAC PE=1 SV=1	DSVSVVLGQHFFN#R	known
Q9HDC9	Adipocyte plasma membrane-associated protein OS=Homo sapiens GN=APMAP PE=1 SV=2	AGPN#GTLFVADAYK	known
Q12841	Follistatin-related protein 1 OS=Homo sapiens GN=FSTL1 PE=1 SV=1	GSN#YSEILDK	prediction
P00736	Complement C1r subcomponent OS=Homo sapiens GN=C1R PE=1 SV=2	CN#YSIR	known
P00736	Complement C1r subcomponent OS=Homo sapiens GN=C1R PE=1 SV=2	EHEAQSN#ASLDVFLGHTNVEELMK	known
Q13822	Ectonucleotide pyrophosphatase/phosphodiesterase family member 2 OS=Homo sapiens GN=ENPP2 PE=1 SV=3	AIIAN#LTCK	prediction
P41222	Prostaglandin-H2 D-isomerase OS=Homo sapiens GN=PTGDS PE=1 SV=1	WFSAGLASN#SSWLR	known
P41222	Prostaglandin-H2 D-isomerase OS=Homo sapiens GN=PTGDS PE=1 SV=1	SVVAPATDGGLN#LTSTFLR	known

P80188	Neutrophil gelatinase-associated lipocalin OS=Homo sapiens GN=LCN2 PE=1 SV=2	SYN#VTSVLFR	known
Q9UHG3	Prenylcysteine oxidase 1 OS=Homo sapiens GN=PCYOX1 PE=1 SV=3	LLHALGGDDFLGMLN#R	known
O14786	Neuropilin-1 OS=Homo sapiens GN=NRP1 PE=1 SV=3	IGYSNN#GSDWK	known
O14786	Neuropilin-1 OS=Homo sapiens GN=NRP1 PE=1 SV=3	RGPECSQN#YTTPSGVIK	known
Q9Y4L1	Hypoxia up-regulated protein 1 OS=Homo sapiens GN=HYOU1 PE=1 SV=1	VIN#ETWAWK	known
Q9Y4L1	Hypoxia up-regulated protein 1 OS=Homo sapiens GN=HYOU1 PE=1 SV=1	LSALDNLLN#HSSMFLK	known
Q9NZP8	Complement C1r subcomponent-like protein OS=Homo sapiens GN=C1RL PE=1 SV=2	N#QSVNVFLGHTAIDEMLK	known
Q02985	Complement factor H-related protein 3 OS=Homo sapiens GN=CFHR3 PE=1 SV=2	FVQGN#STEVACHPGYGLPK	known
O75144	ICOS ligand OS=Homo sapiens GN=ICOSLG PE=1 SV=2	LFN#VTPQDEQK	known
O75144	ICOS ligand OS=Homo sapiens GN=ICOSLG PE=1 SV=2	TVVTYHIPQN#SSLENVDSR	known
P08519	Apolipoprotein(a) OS=Homo sapiens GN=LPA PE=1 SV=1	WEYCN#LTR	prediction
O95866	Protein G6b OS=Homo sapiens GN=G6B PE=1 SV=1	VN#LSCGGVSHPIR	prediction
P28906	Hematopoietic progenitor cell antigen CD34 OS=Homo sapiens GN=CD34 PE=1 SV=2	LTQGICLEQN#K	prediction
Q6UX71	Plexin domain-containing protein 2 OS=Homo sapiens GN=PLXDC2 PE=1 SV=1	VN#LSFDFPFYGHFLR	known
Q9NZK5	Adenosine deaminase CECR1 OS=Homo sapiens GN=CECR1 PE=1 SV=2	NILDALMLN#TTR	known
O76027	Annexin A9 OS=Homo sapiens GN=ANXA9 PE=1 SV=3	TFLN#FSVDK	unknown
Q12884	Seprase OS=Homo sapiens GN=FAP PE=1 SV=5	SVN#ASNYGLSPDR	known

P02786	Transferrin receptor protein 1 OS=Homo sapiens GN=TFRC PE=1 SV=2	DFEDLYTPVN#GSIVIVR	known
P28799	Granulins OS=Homo sapiens GN=GRN PE=1 SV=2	EN#ATTDLLTK	known
P01880	Ig delta chain C region OS=Homo sapiens GN=IGHD PE=1 SV=2	TLLN#ASR	known
P01880	Ig delta chain C region OS=Homo sapiens GN=IGHD PE=1 SV=2	EVN#TSGFAPARPPPQPGSTTFWAWSVLR	known
P16070	CD44 antigen OS=Homo sapiens GN=CD44 PE=1 SV=3	AFN#STLPTMAQMEK	known
Q9UEW3	Macrophage receptor MARCO OS=Homo sapiens GN=MARCO PE=1 SV=1	VDN#FTQNPGMFR	known
Q6UY14	ADAMTS-like protein 4 OS=Homo sapiens GN=ADAMTSL4 PE=1 SV=2	LVSGN#LTDR	known
P48740	Mannan-binding lectin serine protease 1 OS=Homo sapiens GN=MASP1 PE=1 SV=3	FGYILHTDN#R	known
P08571	Monocyte differentiation antigen CD14 OS=Homo sapiens GN=CD14 PE=1 SV=2	N#VSWATGR	known
Q6YHK3	CD109 antigen OS=Homo sapiens GN=CD109 PE=1 SV=2	IN#YTVPQSGTFK	known
Q9H9B1	Histone-lysine N-methyltransferase EHMT1 OS=Homo sapiens GN=EHMT1 PE=1 SV=4	FYGN#VSR	unknown
O94898	Leucine-rich repeats and immunoglobulin-like domains protein 2 OS=Homo sapiens GN=LRIG2 PE=1 SV=3	DFVCDDFLKPQIRTHPETIIALRGMN#VTLTCTAVSSSDSPMSTVWR	prediction
P55056	Apolipoprotein C-IV OS=Homo sapiens GN=APOC4 PE=1 SV=1	MKELLETVVN#R	known
Q9H9J4	Ubiquitin carboxyl-terminal hydrolase 42 OS=Homo sapiens GN=USP42 PE=1 SV=3	VGAGLQNLGN#TCFANAALQCLTYTPPLANYMLSHEHSK	unknown

N# denotes the N-glycosylation sites.

Figure S6. Venn plots of the reproducibility of identified N-glycoproteins a), N-glycopeptides b) and N-glycosylated sites c) in three replicates.

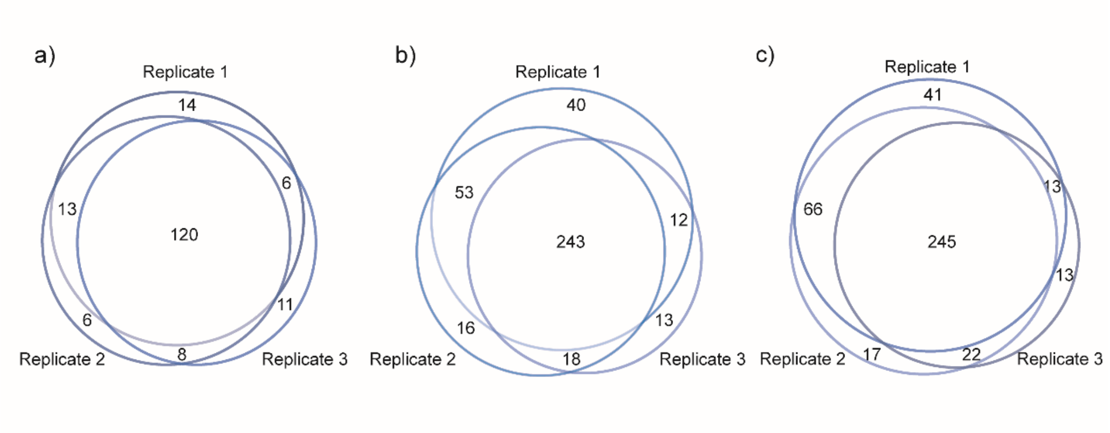

Table S5 List of identified glycoproteins and glycopeptide sequences from tryptic digests of 0.1 μL human plasma enriched by zwitterion functionalized PAMAM and analyzed by only one LC-MS/MS.

Protein accession number	Protein description	Peptide sequence
P00738	Haptoglobin OS=Homo sapiens GN=HP PE=1 SV=1	NLFLN#HSEN#ATAK
P00738	Haptoglobin OS=Homo sapiens GN=HP PE=1 SV=1	VVLHPN#YSQVDIGLIK
P00738	Haptoglobin OS=Homo sapiens GN=HP PE=1 SV=1	MVSHHN#LTTGATLINEQWLLTTAK
P00739	Haptoglobin-related protein OS=Homo sapiens GN=HPR PE=1 SV=2	NLFLN#HSEN#ATAK
P00739	Haptoglobin-related protein OS=Homo sapiens GN=HPR PE=1 SV=2	MVSHHN#LTTGATLINEQWLLTTAK
P0C0L4	Complement C4-A OS=Homo sapiens GN=C4A PE=1 SV=1	GLN#VTLSSTGR
P0C0L4	Complement C4-A OS=Homo sapiens GN=C4A PE=1 SV=1	FSDGLESN#SSTQFEVK
P01011	Alpha-1-antichymotrypsin OS=Homo sapiens GN=SERPINA3 PE=1 SV=2	YTGN#ASALFILPDQDK
P01011	Alpha-1-antichymotrypsin OS=Homo sapiens GN=SERPINA3 PE=1 SV=2	TLN#QSSDELQLSMGNAMFVK

P01011	Alpha-1-antichymotrypsin OS=Homo sapiens GN=SERPINA3 PE=1 SV=2	FN#LTETSEAEIHQSFQHLLR
P02763	Alpha-1-acid glycoprotein 1 OS=Homo sapiens GN=ORM1 PE=1 SV=1	QDQCIYN#TTYLNVQR
P02765	Alpha-2-HS-glycoprotein OS=Homo sapiens GN=AHSG PE=1 SV=1	VCQDCPLLAPLN#DTR
P02765	Alpha-2-HS-glycoprotein OS=Homo sapiens GN=AHSG PE=1 SV=1	AALAAFNAQNN#GSNFQLEEISR
P01042	Kininogen-1 OS=Homo sapiens GN=KNG1 PE=1 SV=2	ITYSIVQTN#CSK
P01042	Kininogen-1 OS=Homo sapiens GN=KNG1 PE=1 SV=2	LNAENN#ATFYFK
P02787	Serotransferrin OS=Homo sapiens GN=TF PE=1 SV=3	CGLVPVLAENYN#K
P02787	Serotransferrin OS=Homo sapiens GN=TF PE=1 SV=3	QQQHLFGSN#VTDCSGNFCLFR
P00450	Ceruloplasmin OS=Homo sapiens GN=CP PE=1 SV=1	AGLQAFFQVQECN#K(S)
P00450	Ceruloplasmin OS=Homo sapiens GN=CP PE=1 SV=1	EHEGAIYPDN#TTDFQR
P00450	Ceruloplasmin OS=Homo sapiens GN=CP PE=1 SV=1	EN#LTAPGSDSAVFFEQGTTR
P00450	Ceruloplasmin OS=Homo sapiens GN=CP PE=1 SV=1	ELHHLQEQN#VSNAFLDKGEFYIGSK
P01023	Alpha-2-macroglobulin OS=Homo sapiens GN=A2M PE=1 SV=3	SLGNVN#FTVSAEALESQELCGTEVPSVPEHGR
P19652	Alpha-1-acid glycoprotein 2 OS=Homo sapiens GN=ORM2 PE=1 SV=2	QNQCFYN#SSYLNVQR
P19652	Alpha-1-acid glycoprotein 2 OS=Homo sapiens GN=ORM2 PE=1 SV=2	QN#QCFYN#SSYLNVQR
P01009	Alpha-1-antitrypsin OS=Homo sapiens GN=SERPINA1 PE=1 SV=3	YLGN#ATAIFFLPDEGK
P01859	Ig gamma-2 chain C region OS=Homo sapiens GN=IGHG2 PE=1 SV=2	EEQFN#STFR
P01859	Ig gamma-2 chain C region OS=Homo sapiens GN=IGHG2 PE=1 SV=2	TKPREEQFN#STFR
P01024	Complement C3 OS=Homo sapiens GN=C3 PE=1 SV=2	TVLTPATNHMGN#VTFTIPANR

P01877	Ig alpha-2 chain C region OS=Homo sapiens GN=IGHA2 PE=1 SV=3	TPLTAN#ITK
P08185	Corticosteroid-binding globulin OS=Homo sapiens GN=SERPINA6 PE=1 SV=1	AQLLQGLGFN#LTER
P01876	Ig alpha-1 chain C region OS=Homo sapiens GN=IGHA1 PE=1 SV=2	LAGKPTHVN#VSVVMAEVDGTCY
P02790	Hemopexin OS=Homo sapiens GN=HPX PE=1 SV=2	SWPAVGN#CSSALR
P04196	Histidine-rich glycoprotein OS=Homo sapiens GN=HRG PE=1 SV=1	VIDFN#CTTSSVSSALANTK
P01008	Antithrombin-III OS=Homo sapiens GN=SERPINC1 PE=1 SV=1	SLTFN#ETYQDISELVYGAK
P01857	Ig gamma-1 chain C region OS=Homo sapiens GN=IGHG1 PE=1 SV=1	EEQYN#STYR
P01861	Ig gamma-4 chain C region OS=Homo sapiens GN=IGHG4 PE=1 SV=1	EEQFN#STYR
P01871	Ig mu chain C region OS=Homo sapiens GN=IGHM PE=1 SV=3	THTN#ISESHPN#ATFSAVGEASICEDDWNSGER
P02749	Beta-2-glycoprotein 1 OS=Homo sapiens GN=APOH PE=1 SV=3	VYKPSAGN#NSLYR
P04004	Vitronectin OS=Homo sapiens GN=VTN PE=1 SV=1	NN#ATVHEQVGGPSLTSDLQAQSK
P10909	Clusterin OS=Homo sapiens GN=CLU PE=1 SV=1	LAN#LTQGEDQYYLR
Q14624	Inter-alpha-trypsin inhibitor heavy chain H4 OS=Homo sapiens GN=ITIH4 PE=1 SV=4	LPTQN#ITFQTESSVAEQEAEFQSPK
P08603	Complement factor H OS=Homo sapiens GN=CFH PE=1 SV=4	MDGASN#VTCINSR
P27169	Serum paraoxonase/arylesterase 1 OS=Homo sapiens GN=PON1 PE=1 SV=3	VTQVYAEN#GTVLQGSTVASVYK
P05543	Thyroxine-binding globulin OS=Homo sapiens GN=SERPINA7 PE=1 SV=2	TLYETEVFSTDFSN#ISAAK

N# denotes the N-glycosylation sites.

Figure S7. Nano-LC-MS/MS mass spectra of doubly charged N-glycopeptide EEQFN#STFR [$(M+2H)^{2+}$ at m/z 579.76] from Ig gamma-2 chain C region a), and QDQCIYN#TTYLNVQR [$(M+2H)^{2+}$ at m/z 958.94] from Alpha-1-acid glycoprotein 1 b). N# denotes the N-glycosylation sites.

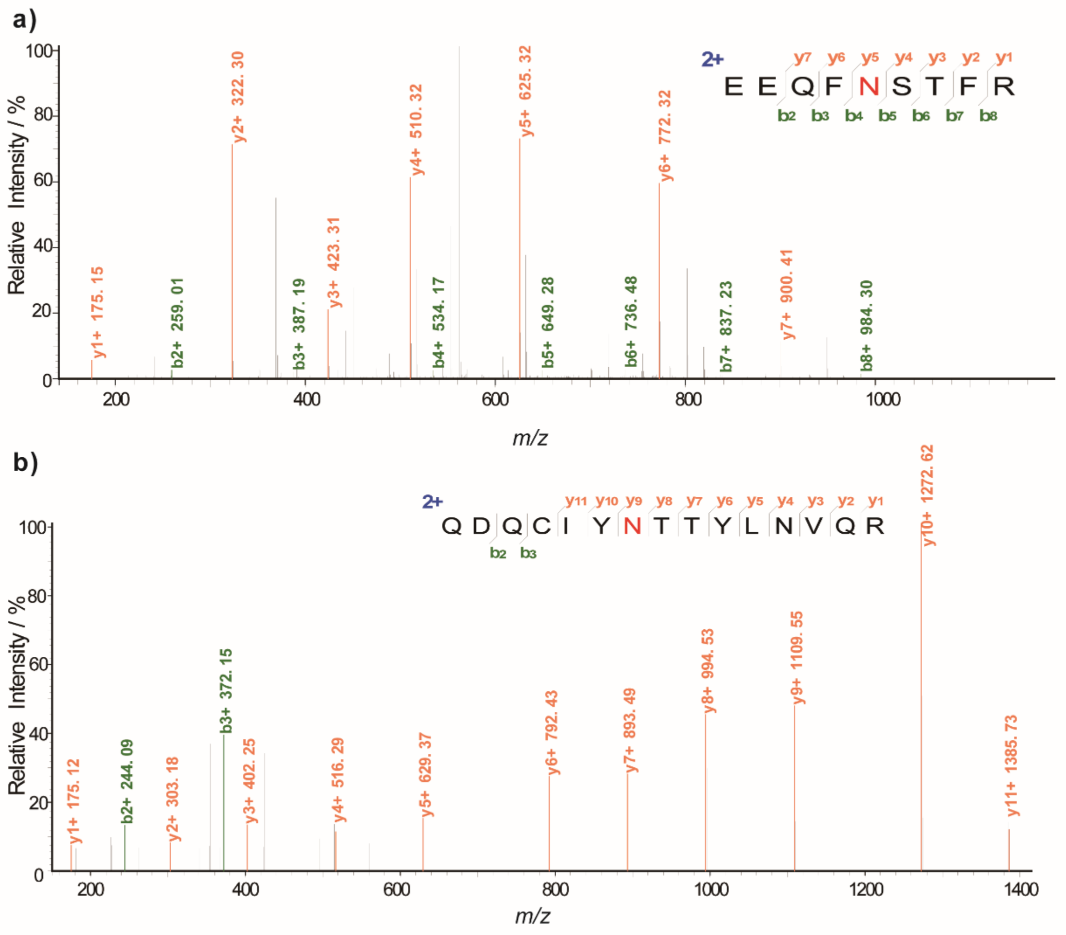

Supplement: Supplementary Information [file srep29776-s1.pdf]
